# RAR-Dependent and RAR-Independent RXR Signaling in Stem-like Glioma Cells

**DOI:** 10.3390/ijms242216466

**Published:** 2023-11-17

**Authors:** Amanda Dabrock, Natalie Ernesti, Florian Will, Manaf Rana, Nadja Leinung, Phillip Ehrich, Volker Tronnier, Christina Zechel

**Affiliations:** 1 Laboratory of Experimental Neuro-Oncology, Center of Brain, Behavior and Metabolism, University Lübeck, Marie-Curie Strasse 66, D-23562 Lübeck, Germany; 2 Department of Neurosurgery, University Clinic Schleswig-Holstein, Campus Lübeck, Ratzeburger Allee 160, D-23538 Lübeck, Germany

**Keywords:** malignant glioma, retinoic acid receptors, retinoid-X receptor, synthetic retinoids, CRISPR/Cas9 editing

## Abstract

Retinoic acid (RA) exerts pleiotropic effects during neural development and regulates homeostasis in the adult human brain. The RA signal may be transduced through RXR (retinoid-X receptor)-non-permissive RA receptor/RXR heterodimers or through RXR-permissive RXR heterodimers. The significance of RA signaling in malignant brain tumors such as glioblastoma multiforme (GBM) and gliosarcoma (GS) is poorly understood. In particular, the impact RA has on the proliferation, survival, differentiation, or metabolism of GBM- or GS-derived cells with features of stem cells (SLGCs) remains elusive. In the present manuscript, six GBM- and two GS-derived SLGC lines were analyzed for their responsiveness to RAR- and RXR-selective agonists. Inhibition of proliferation and initiation of differentiation were achieved with a RAR-selective pan-agonist in a subgroup of SLGC lines, whereas RXR-selective pan-agonists (rexinoids) supported proliferation in most SLGC lines. To decipher the RAR-dependent and RAR-independent effects of RXR, the genes encoding the RAR or RXR isotypes were functionally inactivated by CRISPR/Cas9-mediated editing in an IDH1-/p53-positive SLGC line with good responsiveness to RA. Stemness, differentiation capacity, and growth behavior were preserved after editing. Taken together, this manuscript provides evidence about the positive impact of RAR-independent RXR signaling on proliferation, survival, and tumor metabolism in SLGCs.

## 1. Introduction

Despite extensive basic and clinical research in the last two decades, the therapy of malignant gliomas remains almost unchanged since 2005 and provides only palliation [1,2,3,4]. Therapy failure can be attributed to the histopathological features, the presence of enzymes that repair the lesions introduced by radio- and chemotherapy, detoxifying proteins such as MGMT (O6-methylguanine-DNA methyltransferase), and the presence of tumor cells with features similar to neural stem cells (SLGCs) [5,6,7,8,9]. SLGCs were isolated from higher grade gliomas, including glioblastoma multiforme (GBM) and the gliosarcoma [4,6,7,8,9,10]. Standard GBM and GS therapy consists of surgical intervention followed by fractionated radiation and chemotherapy with the alkylating compound temozolomide [1,4,11,12,13]. The efficacy of TMZ (Temozolomide) treatment is largely related to the expression of MGMT, which in turn is regulated by the methylation of the MGMT gene promoter [1,4,5,6]. Innovative strategies that try to improve the standard therapy assess the impact of the inhibitors of dysregulated oncogenic proteins and pathways or evaluate the potential benefits of immunotherapies [14]. Whether these therapy concepts might improve GBM therapy remains unclear, since GBM resiliency is based on a complex interrelation between molecular and cellular heterogeneity and the hypoxic and metabolic characteristics of the microenvironment. In particular, the specific properties of SLGCs and their adaptive plasticity appear crucial to therapy failure [4,5,6,8,9].

Similar to normal neural stem cells, SLGCs establish a cellular hierarchy consisting of the stem cell and more differentiated progenitors. The glioma stem cell (type I cell) expresses high levels of the pluripotency factor Sox2 (Sex Determining Region Y)-Box 2), which is crucial for the maintenance of stemness and contributes to the malignant phenotype [9,15,16]. Sox2+/CD133− type I cells differentiate into Sox2+/CD133+ progenitors (type II cells), which in turn generate CD133-negative cells with reduced Sox2 expression (type III cells). Further differentiation results in the production of non-SLGCs, which account for the bulk of the tumor cells [6,9,17]. Since the more differentiated GBM and GS cells are more sensitive to the standard therapy than the SLGCs and type I cells in particular [5,6,17], the induction of differentiation might improve the GBM and GS therapy.

Natural components regulating the differentiation of stem cells and progenitors in the neural system include the vitamin A derivative retinoic acid (RA) [18,19]. The RA isomers all-trans RA (atRA) and 9cis RA (9cRA) mediate pleiotropic effects in development and homeostasis. The RA signal is transduced by the transcription factors RAR (retinoic acid receptor) and RXR (retinoid-X receptor), in which 9cRA activates both receptor types and atRA acts solely via RAR [20]. Three distinct RAR and RXR genes are present in the human genome that code for the isotypes RAR α, β, and γ and RXR α, β, and γ, respectively [20]. Importantly, RXR isotypes form heterodimers with multiple partners, which, in the case of the PPAR (peroxisome-proliferator-activated receptor)/RXR heterodimers, are permissive for RXR-selective ligands (rexinoids), whereas others, such as the RAR/RXR heterodimers, require the binding of an agonist to the partner of RXR [20,21,22]. Evidence has been provided that the imbalance in RAR isotype expression and aberrant expression of RA signaling molecules will contribute to brain tumor development [18,23,24], suggesting that the normalization of RA signaling could be beneficial. Indeed, the combination of atRA and vitamin D, or lithium, metformin, and a PPARγ agonist, might improve GBM therapy [25,26]. However, the biological effects of RA on GBM or GS cells are poorly understood. Specifically, whether RA could induce the differentiation of SLGCs or if the potential effects of RA would be transduced by RXR-non-permissive or RXR-permissive heterodimers remains unclear [20].

In the present study, which is a continuation of our previous work [7], we aimed to analyze whether the activation of RAR-dependent or RAR-independent RXR signaling would have an impact on SLGCs from human glioma specimens. First, we compared the responsiveness of six GBM- and two GS-derived SLGC lines to RAR- and RXR-selective agonists. To further decipher the contribution of RAR-dependent and RAR-independent signaling, the T1338-1 subclone, which primarily consisted of type I cells, displayed good responsiveness to retinoids and rexinoids, and lacked the expression of MGMT, was chosen for further experiments. These included CRISPR/Cas9-mediated editing of the three RAR and RXR genes as well as the subsequent characterization of the clones and their responsiveness to retinoids, rexinoids, and TMZ. We show that the functional *knockout* of one or two of the RAR (or RXR) genes is possible in the respective RA-responsive SLGC line and that the degree of stemness is not affected. Moreover, we provide evidence that RAR-dependent RXR signaling can initiate differentiation, whereas RAR-independent RXR signaling rather mediates pro-proliferative and pro-survival effects, which might be associated with SLGC metabolism.

## 2. Results

### 2.1. Distinct SLGC Lines Respond Differently to Rexinoids

**Figure 1:**
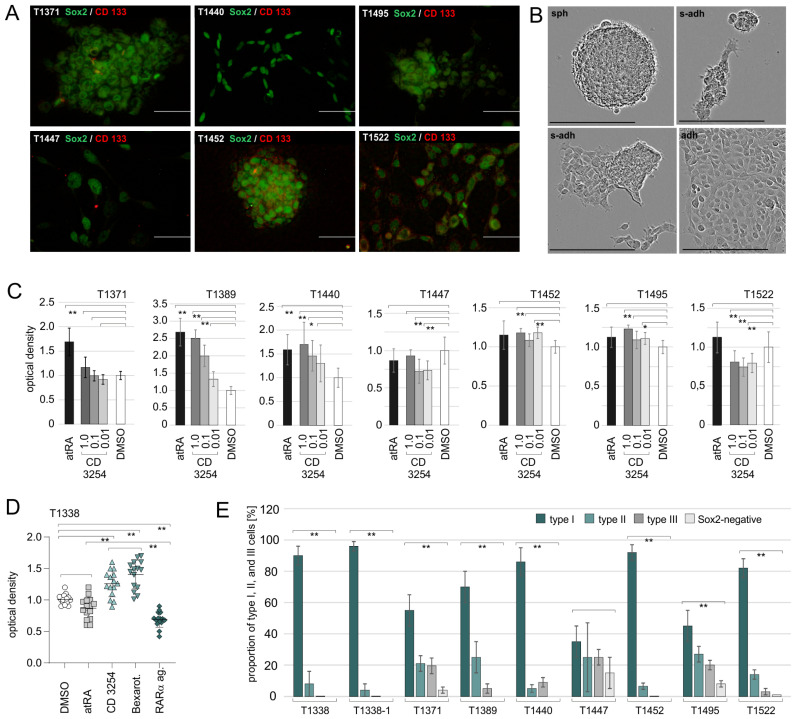
Characteristics of SLGC lines and sensitivity to treatment with rexinoids. (**A**) Phenotypic characterization of SLGC lines: immunocytochemistry analysis (ICC) using the antibody combination mouse-anti-Sox2/rabbit-anti-CD133. Examples of ICCs using a rabbit-anti-Sox2 antibody are shown in the supplementary figures (e.g., Figure S1A–C). Bars, 50 µm. (B) Growth behavior of distinct T1338 cultures. Sph, spherical growth; s-adh, adherent aggregates; adh, adherent growth. Microphotographs were taken by a life-imaging system. Bars, 200 µm. (**C**) BrdU ELISA on day d5 using 1 µM atRA or increasing concentrations of the RXR pan-agonist CD3254 (0.01, 10^−g^ M; 0.1, 10^−M^ M; 1, 10^−M^ M). Statistical comparisons to the DMSO control are shown (*, *p* < 0.05; **, *p* < 0.001). (**D**) Variation of BrdU incorporation in the presence of DMSO, 1 µM atRA, 1 µM of the rexinoids CD3254 or Bexarotene, or 1 µM of the RAR pan-agonist CD0270. The values were determined using 15 biological replicates of T1338 cells and included eight independent values per measurement. For the sake of clarity, only the statistical comparisons to the DMSO control are shown (**, *p* < 0.001). (**E**) Relative abundance of type I, II, and III cells in the SLGC lines analyzed in the present study. The graphs summarize the data from the ICCs of the biological replicates used for the experiments shown in (**C**,**D**). Brackets indicate the statistical comparisons made (**, *p* < 0.001). Sox2, SRY (sex determining region Y)-box 2 (marker for type I, II, and III cells); CD133, Promin-1 (marker for type II cells).

In our previous work, we investigated the responsiveness of SLGC lines to *all-trans* retinoic acid (atRA) and RAR isotype-selective synthetic retinoids [7]. Based on this study, it appeared reasonable that the RXR isotypes might contribute to the retinoic acid-mediated effects in an RAR-independent manner. In the present study, we treated six IDH1-positive SLGC lines from GBMs and two IDH1-positive SLGC lines from GSs [27] with increasing concentrations of the RXR pan-agonist CD3254. All SLGC lines encompassed Sox2-positive cells but differed with respect to the expression of CD133 and their growth behavior (Figure 1A,B, Supplementary Figure S1A–C and File S2 Section S1).

The impact of the rexinoid CD3254 on proliferation was analyzed on days d3, d5, and d7 using BrdU ELISA. Treatment with 1 µM atRA served as a control. The most reliable results were obtained on day d5 (data for d5 are shown in Figure 1C,D) since slow growth rates on d3 or overgrowth on d7 were observed in some cases, which limited the interpretability of the respective results. In the cases of three SLGC lines (T1371, T1389, and T1440), 1 µM atRA significantly increased BrdU incorporation, whereas four SLGC lines (T1447, T1452, T1495, and T1522) showed no significant effects (Figure 1C). The rexinoid CD3254 mediated a small but non-significant pro-proliferative effect on T1371 and significant pro-proliferative effects in the cases of T1389, T1440, T1452, T1495, and T1338 (Figure 1C,D). A significant CD3254-mediated reduction in proliferation was deduced for T1447 and T1522 (Figure 1C). The responsiveness to atRA or the rexinoid appeared independent of the degree of stemness, since the relative numbers of type I, II, and III cells were not related to the ligand’s impact on proliferation. For example, T1338 (or T1452) and T1522 cultures, which contained the highest relative numbers of type I cells (Figure 1E), displayed opposite behavior after treatment with CD3254. A similar contrary responsiveness was observed for T1495 and T1447, both of which establish a cellular hierarchy with similar relative numbers of type I, II, and III cells (Figure 1E and Figure S1A–C). When the rexinoid CD3254 and the RAR pan-agonist CD0270 were analyzed together, the effects on BrdU incorporation were very similar for CD0270/CD3254 (A/X in Figure S1D) and CD0270 alone, in the cases of T1440, T1447, and T1495. Only in the case of T1371, the optical densities observed for CD0270/CD3254 (1 and 0.1 µM CD0270) were significantly lower than those for CD0270 and CD3254 alone (Figure S1D). Taken together, these data suggest that the activation of RXR isotypes barely enhanced the RAR-mediated effects and that RAR-independent activation of RXR signaling has a pro-proliferative effect in most SLGC lines.

The degree of stemness (File S2 Section S2), which was deduced from the expression of the transcription factor Sox2 and the glycoprotein CD133 [15,16,17], was only affected by CD0270 and/or CD3254 in one (T1338) out of six GBM-derived SLGC lines and none of the GS-derived SLGC lines (examples in Figure S2). In addition, neural differentiation (File S2 Section S3) was only evident for T1338. In brief, the induction or upregulation of the glial fibrillary protein GFAP [28] was observed for T1338, and a few single GFAP-positive cells were induced by atRA and the rexinoid in the case of T1371 (examples in Figures S2 and S3B). Moreover, the expression of the microtubule-associated protein Tau [29], which is already detectable in all SLGC lines even in the presence of a high level of Sox2 expression, was upregulated in T1338 cultures. Notably, the pan-RAR agonist supported the upregulation of Tau and the pan-RXR agonist, the upregulation of GFAP in T1338 cultures (examples in Figure S3B).

### 2.2. T1338-1 Cells Express All Three RAR and RXR Isotypes

**Figure 2:**
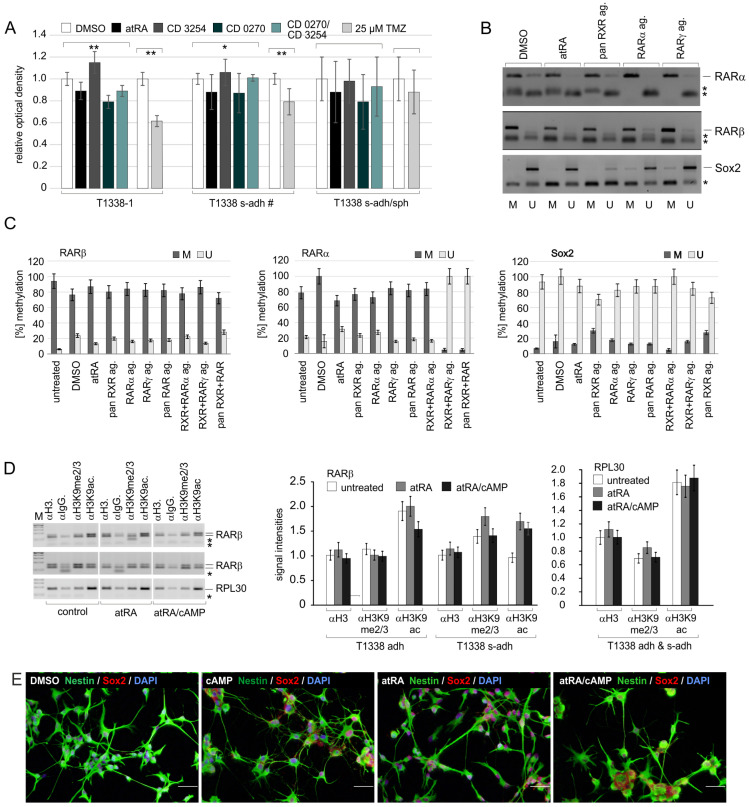
Characteristics of T1338 subclones. (**A**) Responsiveness of T1338 subclones to retinoids and TMZ (temozolomide). BrdU incorporation in the presence of DMSO, 1 µM atRA, or 1 µM CD3254, the RAR pan-agonist CD0270 or CD0270/CD3254. The values were determined using three biological replicates and included eight independent values per replicate. Statistical comparisons are indicated by brackets (*, *p* < 0.05; **, *p* < 0.001). s-adh #, T1338-2 and -3; s-adh/sph, T1338-9. (**B**,**C**) Methylation status of the RARβ, RARα, or Sox2 promoter and impact of ligands. M, methylated; U, unmethylated. The agarose gels (panel **B**) depict one example of four biological replicates of T1338-1; the symbol * indicates the position of oligo-nucleotides and/or unspecific PCR products. The graphs (panel **C**) summarize the data from these replicates. Mean values and standard deviations are shown. (**D**) Chromatin-IP indicating the posttranslational modifications of histone H3 in the RARβ gene promoter: H3K9ac, active mark; H3K9me2/3, repressive mark; ac, acetylated; me2/3, di-/tri-methylated. The agarose gels (**left** panel) depict the results of two independent biological replicates; the symbol * indicates the position of oligo-nucleotides and/or unspecific PCR products. adh, T1338-6; s-adh, T1338-1 and -2. The bars in the graphs summarize the data from three technical replicates. The IPs with the IgG antibody served as negative controls. (**E**) Analysis of the differentiation capacity of the biological replicate of T1338-1 used as the mother culture for the editing approaches. Immunocytochemistry analysis using antibodies against Nestin and Sox2 (SRY (sex determining region Y)-box 2) and nuclear counterstain with DAPI (4’,6-Diamidino-2-phenylindol). Bars, 50 µm.

The informative value of experiments using synthetic RAR- or RXR-selective ligands is limited by their partially unfavorable K_D_s and low specificities [7], and rexinoids with a satisfactory RXR isotype selectivity are not available. Therefore, we decided to apply CRISPR/Cas9-mediated editing to evaluate the potential roles of RAR and RXR isotypes for SLGC stemness, proliferation, differentiation, and responsiveness to TMZ. The above data indicated that the T1338 SLGC line would be more suitable for these analyses than other SLGC lines, since it contained primarily type I cells and showed good responsiveness to synthetic ligands. Due to the heterogeneity of the original T1338 culture with regard to the p53 status, limited dilution assays were necessary to isolate a clone with a pure p53 wildtype (WT) status (for details, see Supplementary File S2 Section S4). In BrdU ELISA, the isolated p53^WT/WT^ clones displayed differences in their responsiveness to retinoids and rexinoids and TMZ, which appeared to be related to their growth behavior (Figure 2A).

To be able to use the most suitable T1338 p53^WT/WT^ clone and to interpret the editing experiments, we had to analyze several critical parameters beforehand, which included the expression of the various RAR and RXR isotypes and the capacity for differentiation (for details, see Supplementary File S2 Section S4). In this connection, RARα and particularly RARβ deserved specific attention since some reports suggested a crucial role for RARα and RARβ dysregulation in GBM cells [18,23,24].

Western blot, qRT-PCR, and immunocytochemistry analyses confirmed the expression of all three RAR and RXR isotypes in T1338-1 (Figure S3C–E). MSP analyses (Figure 2B,C) indicated a high degree of methylation of the RARβ gene promoter, which remained largely unchanged after treatment with atRA, RAR-selective agonists, the rexinoid CD3254, or combinations of RAR-selective agonists and CD3254 (Figure 2C, left). In contrast, methylation of the RARα gene promoter became reduced after co-stimulation of RAR and RXR signaling (Figure 2C, middle). In keeping with the immunocytochemistry analyses investigating the stemness state, the Sox2 gene promoter was hypomethylated in the T1338-1 subclone, and atRA and the synthetic retinoids induced only minor changes (Figure 2C, right). To further clarify the status of the RARβ promoter, we carried out ChIP analyses using antibodies directed against modifications in the lysine residue 9 of histone H3 (H3K9). This decision was based on publications indicating that di- and tri-methylation of H3K9 by several SET-domain methyltransferases might be crucial in the development and treatment of several types of cancer, including malignant brain tumors [30,31,32]. The immunoprecipitations using chromatin from the distinct T1338 subclones and the antibodies against H3K9ac (a histone mark indicating activation) and H3K9me2/3 (a histone mark indicating repression) revealed a certain heterogeneity of the H3K9 status in the RARβ promoter. The T1338 subclones with the s-adh phenotype (including T1338-1) displayed similar degrees of H3K9ac and H3K9me2/3, whereas the T1338-adh clone exhibited higher levels of H3K9ac (Figure 2D). In both cases, however, a reduction of H3K9 methylation by atRA or atRA/cAMP was not evident (Figure 2D, middle). In comparison to the RLP30 promoter, which is active in all cells and serves as a control, the chromatin of the RARβ gene promoter appeared more closed (Figure 2D, right). Taken together, our data indicate that the subclone T1338-1 was suitable for further studies because of its good responsiveness to retinoids, rexinoids, and TMZ, the expression of all three RAR and RXR isotypes, and its capacity to differentiate.

### 2.3. T1338-1 Clones with RAR Knockouts Maintain Proliferation, Stemness, and the Capacity to Differentiate

**Figure 3:**
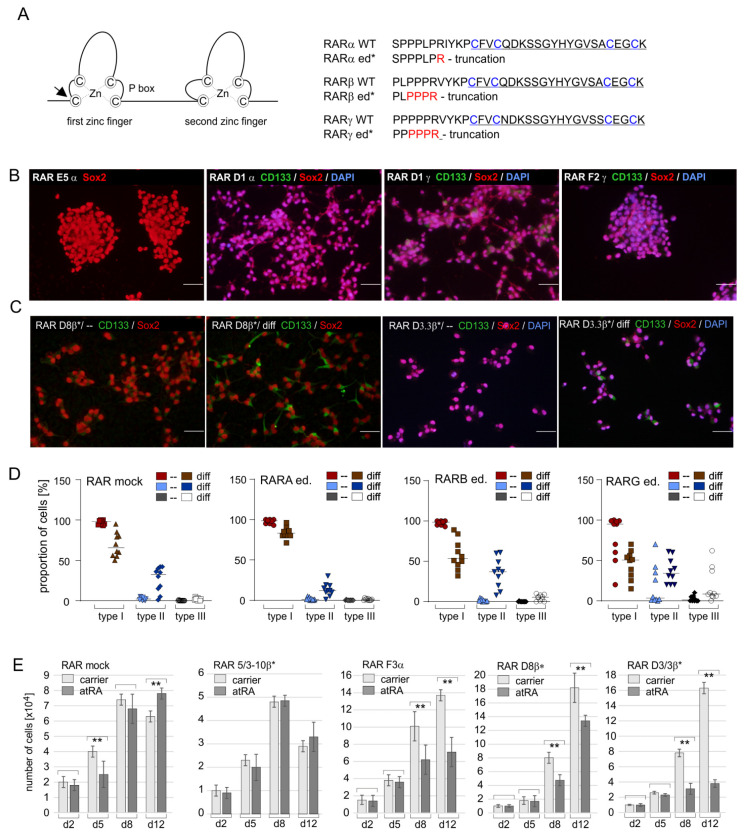
Impact of CRISPR/Cas9-mediated editing of the RAR genes. (**A**) **Left**: schematic drawing indicating the two zinc fingers of the RAR DBDs and the P box (-**C**EG**C**K-). The black arrow indicates the position of editing. **Right**: amino acid sequences of the first zinc finger (underlined) of RARα, β, and γ; zinc complexing cysteine residues are shown in blue. In general, the editing process eliminated the amino acids C-terminally of the residues indicated in red (for details, see Figures S4, S5 and S6A–C). WT, wildtype; ed*, edited clones. (**B**) Immunocytochemistry analyses of edited RAR clones using antibodies against Sox2 and CD133. DAPI nuclear counterstain was omitted from the panel RAR E5α. Bars, 50 µm. (**C**) Similar analysis as in (**B**) investigating the differentiation capacity of the clones indicated. Bars, 50 µm. (**D**) Quantification of the presence of type I, II, and III cells in proliferating clones and after induction of differentiation with a RAR pan-agonist. Data from mock controls and RAR clones with the same type of editing (ed.) were combined. Mean values are indicated by a black line. (**E**) Growth curves of three edited clones and one mock-edited clone in the presence of 1 µM atRA or the carrier DMSO. Cell numbers were determined on days d2, d5, d8, and d12. The mean values of four independent assays are shown; whiskers indicate standard deviations; statistical comparisons are indicated by brackets (**, *p* < 0.001). For the sake of clarity, only statistical comparisons between the treated and untreated samples from the same day are shown. β* indicates monoallelic editing of the RARB gene.

Since the efficacy of standard transfection techniques is very low for SLGCs, we applied a lentiviral system for CRISPR/Cas9-mediated editing [33,34]. In all three cases, the single-guide RNA was directed against the sequence coding for the N-terminal part of the respective first zinc fingers (Figure 3A left; for details, see File S2 Section S5). In the first approach, either the RARA, the RARB, or the RARG genes were targeted. The second round of editing aimed to generate functional knockouts in two RAR isotype genes.

It transpired that targeting of the RARα gene was efficient and resulted in the insertion of one single nucleotide four bases at 5’ of the PAM sequence NGG in most clones (Figures S4A and S5). Moreover, this frameshift mutation was homozygous, and the inserted nucleotide was an adenosine monophosphate in >95% of these cases. Due to this frameshift mutation, most RAR clα clones constitute full RARα knockouts (Figure 3A right and Figure S6A). The efficiency of editing the RARβ and RARγ genes was much lower as it generated highly heterogeneous mutations, and residual wildtype sequences were present in several clones (examples in Figures S4B, S5, and S6A). To isolate RAR clβ and RAR clγ clones with biallelic editing, additional rounds of limited dilution assays were necessary. Finally, one RARβ clone with a biallelic knockout, three RARβ clones with a monoallelic knockout, and three RARγ clones with a biallelic editing were isolated (Figures S4, S5, and S6A,B). In this context, it should be noted that the knockout of two distinct RAR isotypes was possible in T1338-1 cells (File S2 Section S5; Figure S5).

Immunocytochemistry analyses using antibodies against Sox2 and CD133 indicated that the stemness state was preserved during editing (examples in Figure 3B–D). With the exception of one RARG clone (RAR F2γ), the cultures of all edited RAR clones primarily encompassed type I cells and differentiation could be induced with a RAR pan-agonist (Figure 3B,C and quantification in Figure 3D). The capacity of the edited RAR clones to undergo neural differentiation after retinoid treatment was investigated using immunocytochemistry. These analyses included two mock-edited clones, which were isolated from an editing experiment that was performed in parallel and used the original lenti-CRISPR v2 vector. The RAR pan-agonist CD1556, but not the RARβ-agonist CD1886, increased the relative numbers of type II versus type I cells in the RAR clones and mock controls (examples in Figure 3C). The upregulation of GFAP and/or Tau was detectable after treatment with the RAR pan-agonist (examples in Figure S6D). Upregulation of CD133, or GFAP, and Tau was observed in all RAR clone cultures and the mock controls, though with low efficacy in most cases (Figure 3D). Only minor changes became evident in these assays, which was expected since the cultures were exclusively treated with the retinoid and not with a combination consisting of retinoid plus cAMP. Retinoids are less potent inducers of neural differentiation in the absence of cAMP as compared to 1 µM atRA/1 mM cAMP [35,36]. However, to exclude any bypassing of the RA signal [37], cAMP was not added in these experiments.

The potential impact of atRA on the RAR clones was additionally investigated using growth curves. Foremost, these growth curves showed that the proliferation of the clones was not compromised by the biallelic or monoallelic knockouts of the RAR genes. The responsiveness to 1 µM of atRA, however, varied not only between the clones but also between biological replicates of the same clone (examples in Figure 3E). This may be explained by the variations in growth behavior and the size of the aggregates. Moreover, T1338 aggregates detach from the growth substrate and reattach at a distinct position when analyzed under the conditions applied to growth curves. Furthermore, T1338 aggregates fuse and release single cells, which enter distinct aggregates (File S2 Section S1). Finally, it has to be mentioned in this context that atRA preparations may undergo spontaneous isomerization to 9cis RA in cell culture medium [38], so that pro-proliferative effects might have been induced by RAR-dependent and RAR-independent RXR signaling.

### 2.4. CRISPR/Cas9-Mediated Editing of RXRA but Not RXRB and RXRG Is Highly Efficient

**Figure 4:**
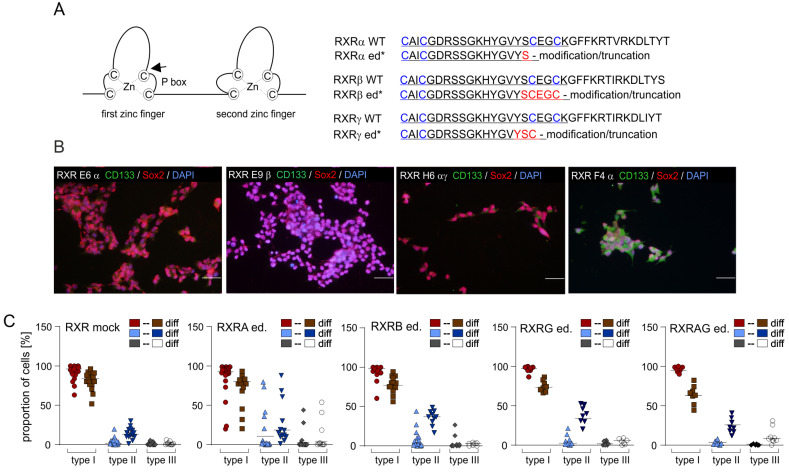

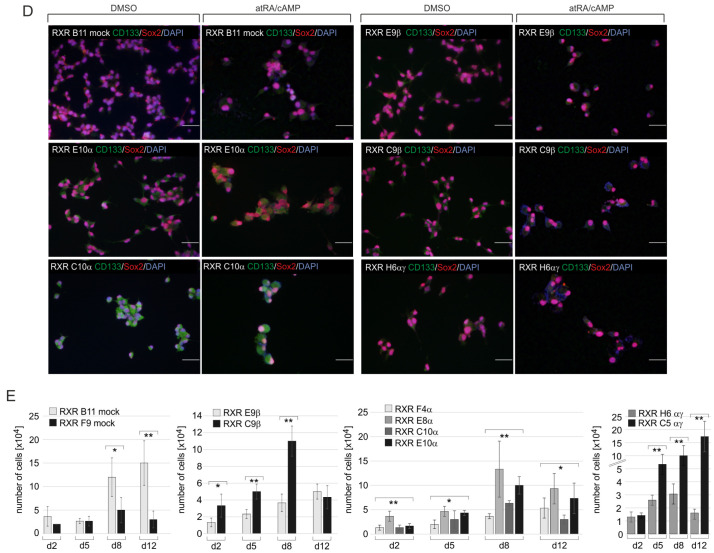
Impact of CRISPR/Cas9-mediated editing of the RXR genes. (**A**) **Left**: schematic drawing indicating the two zinc fingers of the RXR DBDs and the P box (-**C**EG**C**K-). The black arrow indicates the position of editing. **Right**: amino acid sequences of the first zinc finger (underlined) of RXRα, β, and γ; zinc complexing cysteine residues are shown in blue. In general, the editing process eliminated the amino acids C-terminally of the residues indicated in red (for details, see Figures S7 and S8A–C). (**B**) Immunocytochemistry analyses of the stemness state and DAPI nuclear counterstain. Bars, 50 µm. (**C**) Quantification of the presence of type I, II, and III cells in edited clones (ed.) and impact of the induction of differentiation (diff). Mock controls and RXR clones with the same type of *knockout* are summarized in one graph each. Data from 2–3 biological replicates are combined. Mean values are indicated by a black line. (**D**) A similar experiment as in (**B**) investigating the capacity for differentiation. Bars, 50 µm. (**E**) Growth curves of seven edited and two mock clones. Cell numbers were determined on days d2, d5, d8, and d12. The mean values of five independent replicates are shown; whiskers indicate standard deviations. Statistical comparisons are indicated by brackets (*, *p* < 0.05; **, *p* < 0.001). For the sake of clarity, only statistical comparisons between the samples from the same day are shown.

The single guide RNAs used for the targeting of the RXR isotype genes were directed against the sequence encoding the C-terminal part of the respective first zinc fingers (Figure 4A; for details, see Figure S7). Apart from that, the RXR editing approaches followed a similar strategy as the afore RAR editing approaches, i.e., after an initial editing of only one RXR isotype gene, a second round of editing was undertaken, which aimed to simultaneously knockout two RXR isotype genes in the same cell. Clones that did not harbor any modifications in the RXRα, RXRβ, or RXRγ genes were classified as “RXR mock” clones. Editing of the RXRα gene was efficient and resulted in several clones with a homozygous insertion of a thymidine monophosphate with four nucleotides at 5′ of the PAM (e.g., RXR E8α). In addition, several RXRα clones carried two distinct mutations (e.g., RXRA D5α and RXRA E10α) or more than two mutations (RXRA F4α), suggesting that the latter cell clones encompassed more than one cell population (Figures S7 and S8A). The editing of the RXRβ and RXRγ genes generated several clones with monoallelic and a few clones with biallelic editing (Figure 4A, Figures S7 and S8B,C). Erroneous editing of RXRA, RXRG, or RXRG by RXRα-, β-, and γ-specific single guide RNAs did not occur.

The simultaneous editing of (i) RXRα and γ, (ii) RXRα and β, and (iii) RXRβ and γ, respectively, resulted in a substantial amount of clones without any editing. Two clones with double knockouts (RXR C5αγ and RXR H6αγ) were identified. Further analyses revealed that the clone RXR C5αγ carried a homozygous insertion of a thymidine monophosphate in the RXRα gene but was not fully edited in the RXRγ alleles (Figure S7D). On the contrary, the clone RXR H6αγ appeared fully edited in both the RXRα gene (homozygous T insertion) and the RXRγ gene (multiple mutations), suggesting that the culture contained at least two types of edited clones. To isolate pure RXR H6αγ subclones with a homozygous or heterozygous biallelic editing of RXRG, additional rounds of limited dilution assays were necessary. Finally, several RXR H6αγ subclones were obtained that appeared suitable for further analysis (Figure S7D).

### 2.5. The Knockout of a Single RXR Isotype Does Not Affect Proliferation or Stemness

Except for the clones RXR C10α and RXR F4α, the cultures of the RXR clones primarily encompassed type I cells (Figure 4B,D and Figure S8D, and quantification in Figure 4C), indicating a good preservation of stemness. Since all biological replicates of the clones RXR C10α and RXR F4α contained substantial amounts of CD133+/Sox2+ type II cells, this suggests that editing occurred in a type II cell in these cases.

**Figure 5:**
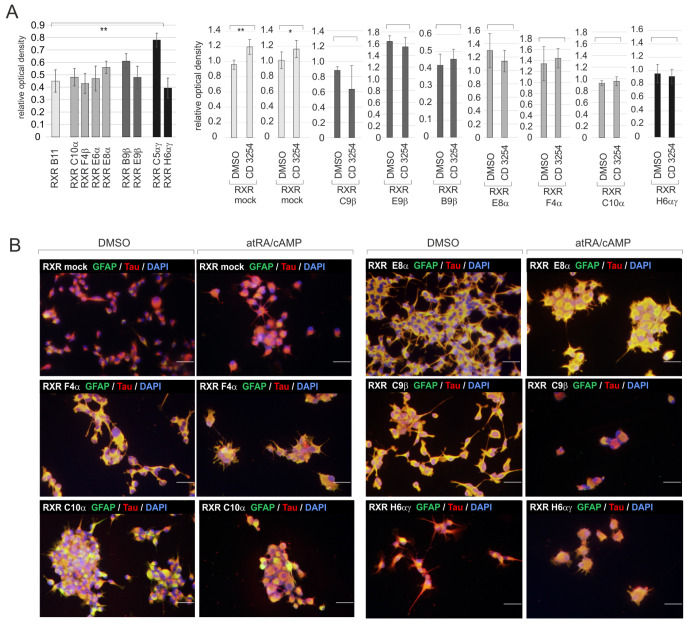
Characteristics of clones with edited RXR genes. (**A**) BrdU-ELISA on day d5: comparison of eight edited clones and one mock clone (B11) and impact of the treatment with 1µM of CD3254. The mean of eight independent values and the standard deviations are shown. The brackets indicate statistical comparisons (*, *p* < 0.05; **, *p* < 0.001). (**B**) Immunocytochemistry analyses using the antibody combination GFAP/Tau and DAPI nuclear counterstain. Expression after treatment with 1 µM atRA/1 mM cAMP and in the corresponding controls. Bars, 50 µm. cAMP (cyclic adenosine monophosphate).

The comparison of the growth curves prepared for the various RXR clones revealed significantly different proliferation rates between RXR clones with the same type of editing, which included the RXR mock clones (examples in Figure 4E). Whether the drastic difference between the growth curves of the clones RXR C5αγ and RXR H6αγ (Figure 4E, right) would reflect the presence of one wildtype RXRγ allele in RXR C5αγ relative to the full knockout of both RXRα and RXRγ in the clone RXR H6αγ remains unclear. The direct comparison of the proliferation of the two respective clones using BrdU ELISA also indicated a higher proliferation rate of RXR C5αγ (left panel in Figure 5A). Further BrdU ELISA (Figure 5A) indicated that the proliferation of the clones with either a RXRα or a RXRβ knockout was similar among each other and to the RXR mock control B11. Furthermore, the RXR pan-agonist CD3254 significantly increased BrdU incorporation, but not in the edited clones.

When treated with 1 µM atRA and 1 mM cAMP, the cell numbers decreased in the cultures of the RXR clones and the mock controls (Figure 5B, Figures S9 and S10). Though visible, a clear induction of CD133 could not be observed, suggesting that a differentiation shift of type I cells toward type II cells did not occur (Figure 5B and Figure S9). Interestingly, the basal levels of GFAP and Tau were already higher in the cases of RXRE10α, RXR E8α, RXR F4α, RXR C10α, RXR H6αγ, and RXR C9β than in the controls (Figure 5B and Figure S10). This suggests that the functional knockout of the RXR isotypes might result in higher expression of the neural proteins GFAP and Tau without the need for a shift to a lower degree of stemness. The treatment with atRA/cAMP further increased the levels of Tau and reduced the cell numbers (Figure S10).

### 2.6. Expression of the Lactate Dehydrogenase A (LDHA) Was Impaired in RXRA/RXRG Double Knockouts

**Figure 6:**
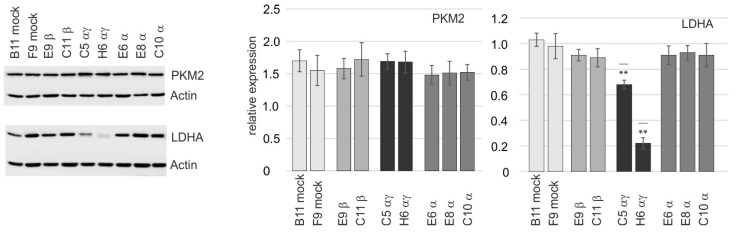
Impact of RXR editing on the expression of metabolic enzymes. Expression of the pyruvate kinase isoform PKM2 and the lactate dehydrogenase isoform A (LDHA) in RXR clones. **Left**: an example of Western blot analysis; the loading control Actin is shown below. **Middle** and **right**: The graphs depict the quantification of the relative expression of the PKM2 and the LDHA, respectively. Significantly distinct expression levels are indicated by ** (*p* < 0.001).

The growth curves and the BrdU ELISA discussed above indicated a different behavior of the RXR clones with a full knockout (RXR H6αγ) or a partial double knockout (RXR C5αγ). Since RXR forms heterodimers with several nuclear receptors that regulate metabolic pathways and are permissive for activation by rexinoids [21], it appeared possible that the functional knockouts of RXR isotypes would impact on, e.g., glycolytic enzymes. Particularly, the pyruvate kinase 2 (PKM2) and the lactate dehydrogenase isoform A (LDHA) appeared interesting since both enzymes are important for the metabolic adaptations observed in cancer cells [39,40]. Indeed, the expression of the PKM2 was high in the mock-edited clones and appeared unchanged after editing of the RXR isotype genes or treatment with the rexinoid bexarotene (Figure 6 and Figure S11). On the contrary, the expression of the LDHA was reduced in the double edited clones with a partial (RXR C5αγ; 30–35% reduction) or full (RXR H6αγ; 80% reduction) RXRγ knockout (Figure 6).

### 2.7. Co-application of the Rexinoid Bexarotene Does Not Improve TMZ Responsiveness

**Figure 7:**
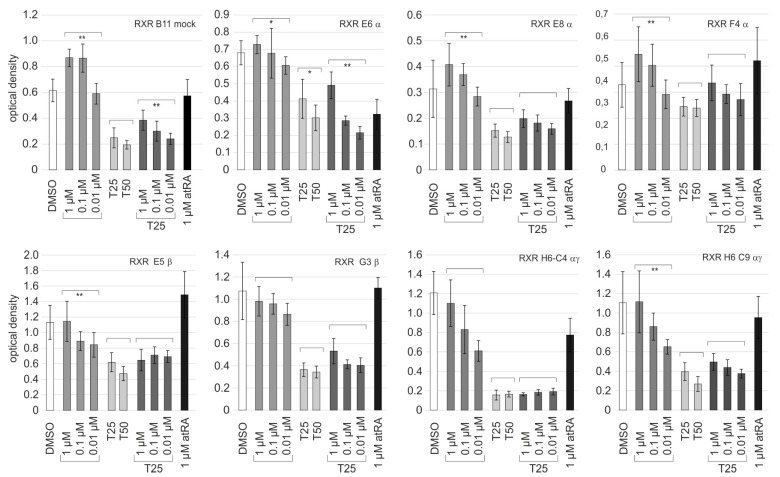
Impact of treatment with TMZ in the presence of bexarotene. BrdU-ELISA on day d5: Comparison of the proliferation of seven edited clones and one mock-edited clone (B11) after treatment with increasing concentrations of bexarotene (1 µM, 0.1 µM, and 0.01 µM) in the absence (light gray bars) and presence (dark gray bars) of 25 µM TMZ (T25). Treatment with 1 µM atRA (black bars), TMZ (T25, 25 µM; T50, 50 µM), or the carrier DMSO (white bars) served as controls. The mean of eight independent values and the standard deviations are shown. For the sake of clarity, only statistical comparisons between the samples from the same treatment groups are shown (*, *p* < 0.05; **, *p* < 0.001).

**Figure 8:**
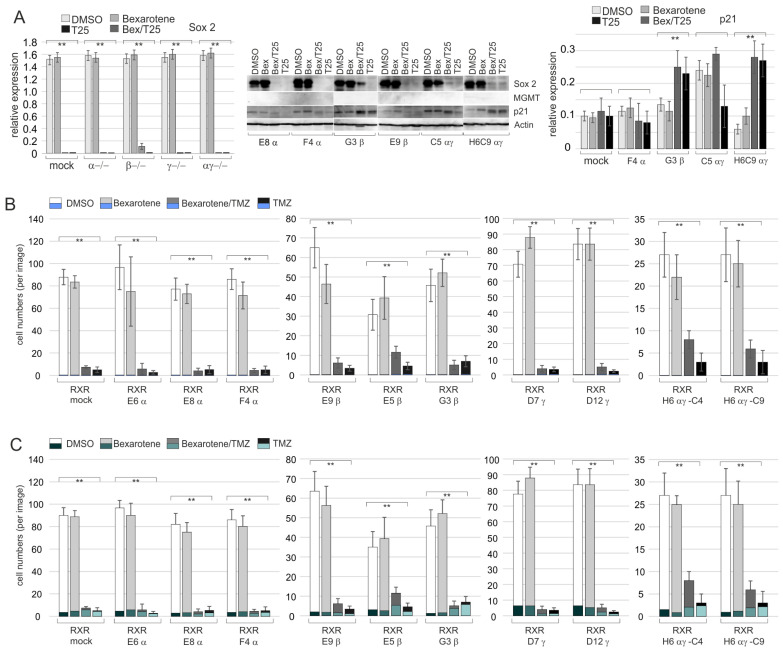
Impact of the treatment with TMZ and Bexarotene on cell numbers and stemness. (**A**) Relative expression of Sox2 (or p21^CIP1^ or MGMT) in clones with edited RXRα (α−/−; E6α, E8α, E10α, F4α), RXRβ (β−/−; E5β, G3β, E9β), RXRγ (γ−/−), as well as clones with no editing (mock) and a double *knockout* (RXRαγ−/−; H6, H6C9, H6C4); the impacts of 25 µM TMZ and 1 µM bexarotene are shown. A representative example of a Western blot is depicted in the middle. Expression of p21 (p21^CIP1^) was low and varied between replicates of the same clones and clones with the same type of *knockout*. Brackets indicate the statistical comparisons performed (**, *p* < 0.001). (**B**,**C**) Quantification of the cell numbers revealed by immunocytochemistry analyses using the antibody combinations CD133/Sox2 (**A**) or Nestin/γH2AX (**B**). The mean values and standard deviations are shown. Brackets indicate the statistical comparisons performed (**, *p* < 0.001). In (**B**), the relative amounts of Sox2-negative cells are indicated in blue, and in (**C**), the relative proportion of γH2AX-positive cells is indicated in green. A robust quantification of type I, II, and III cells was not possible because of staining artifacts in TMZ-treated cell batches.

Finally, we evaluated if the effects mediated by temozolomide (TMZ) would be modulated by the co-application of rexinoids in the RXR-edited clones or the mock controls. Since the rexinoid CD3254 (K_D_ 50 nM for RXRα, RXRβ, and RXRγ) has less favorable K_D_s than the rexinoid bexarotene (K_D_ 24 nM RXRβ, 25 nM RXRγ, 33 nM RXRα), these experiments were performed with the latter ligand. The parameters of proliferation and induction of lesions in DNA were addressed, and the expression levels of Sox2, MGMT, and p21^CIP1^ were determined in parallel (Figure 7, Figure 8 and Figure S12).

In BrdU ELISA, the pro-proliferative effect of bexarotene was statistically significant for all RXR cl-α clones and the RXR mock controls. Since a pro-proliferative effect of 1 µM bexarotene was not evident for clones with edited RXRβ genes and the double knockout (examples in Figure 7), this could suggest that the bexarotene effects were primarily mediated by RXRβ or RXRγ signaling. TMZ (25 µM) reduced the optical densities in all BrdU ELISAs, in which the highest efficacy was observed for the double knockouts. The optical densities determined for the co-application of TMZ and bexarotene could suggest that 1 µM bexarotene might exert a pro-survival effect in some cases (e.g., RXR B11 mock, RXR E8α, RXR F4α, RXR G3β, or RXR H6C9αγ). The differences, however, were small and close to the ranges of experimental variations (Figure 7).

Western blot analyses revealed that bexarotene did not affect the expression of Sox2, nor did it initiate the expression of the repair enzyme MGMT (O6-Methylguanine-DNA Methyltransferase) in the edited clones or controls (Figure 8A). Treatment with 25 µM TMZ almost abrogated the expression of Sox2, whereas the MGMT gene remained silenced (Figure 8A); the latter aspect was additionally verified by MSP assays, which we did not include in the figures. The co-application of TMZ and bexarotene had the same impact on Sox2 and MGMT expression as the sole treatment with TMZ (Figure S8).

A low expression level of the cell cycle inhibitor p21^CIP1^ was observed in all RXR clones and the mock controls (Figure 8B). The majority of these western blots (examples in Figure S8, middle) indicated that bexarotene did not upregulate the p21^CIP1^ levels in the RXR clones or the mock controls. When treated with 25 µM TMZ, an increase in the p21^CIP1^ expression level was observed in some but not all RXR clones. Moreover, the p21 signal appeared as a double band, the cause of which is not known. With a few exceptions, the signal intensity was very similar for the TMZ- and TMZ/bexarotene-treated samples, and clones with the same type of RXR knockout behaved differently. Altogether, this supports the conclusions that the rexinoid bexarotene is not an inhibitor of T1338-1 proliferation and that bexarotene does not intensify TMZ-mediated effects.

Immunocytochemistry analyses (examples in Figure S12A,B) revealed a drastic loss of cells after treatment with 25 µM TMZ (Figure 8B,C). This was less pronounced in the presence of bexarotene in the case of the clones RXR E9β, RXR E5β, RXR D12γ, RXR H6 αγ-C4, and RXR H6αγ-C9, suggesting that bexarotene might partially compensate TMZ-induced cell death. Except for the RXR double knockouts RXR H6 αγ-C4 and RXR H6αγ-C9, these effects were low. Moreover, a correlation between the type of RXR knockout and the beneficial effect of bexarotene did not become evident.

As described above, all RXR clones (except RXR C10α and RXR F4α) exhibited a high degree of stemness and consisted of >90% type I cells (see, e.g., Figure 4). This was not changed after the treatment with bexarotene, and Sox2-negative cells (non-SLGCs) were absent. After the treatment with 25 µM TMZ, a low proportion (less than 5%) of the cells appeared Sox2-negative in the case of the clones RXR E5β, RXR D7γ, and RXR D12γ (Figure 8B and examples in Figure S12A). A quantification of type I, II, and III cells could not be performed because of the staining artifacts in the few surviving cells and the substantial amounts of cell debris in the TMZ-treated batches.

To elucidate whether bexarotene might counteract the cytotoxic effects of TMZ, we aimed to investigate the phosphorylation of the histone variant H2AX, which can be observed in GBM cells after treatment with TMZ [41]. Low numbers of γH2AX+ cells were observed in the DMSO controls and after treatment with bexarotene in the edited RXR clones and the RXR mock controls. Five days after rexinoid treatment, the proportion of γH2AX+ cells was very similar in the controls and the rexinoid-treated cultures (Figure 8C and Figure S12B). On the contrary, >95% of the few cells that survived TMZ treatment displayed γH2AX signals, though with distinct signal strength (examples in Figure S12B). The presence of 0.1 µM bexarotene did not significantly modify the TMZ-mediated effects (Figure 8C), suggesting that the capacity of TMZ to introduce lesions in DNA is not weakened by bexarotene.

## 3. Discussion

Evidence has accumulated that an imbalance of retinoic acid (RA) metabolism and RAR signaling might contribute to the initiation and progression of malignant primary brain tumors [18,23,24]. Moreover, the sequestration of RA in the cytoplasm of GBM cells by the cellular retinoic acid-binding protein 2 (CRABP2) appeared to be related to the poor survival of GBM patients [42]. A very recent study comparing data from The Cancer Genome Atlas (TCGA) indicated that pathways associated with RA metabolism may play an important role in glioma etiology, suggesting that the future exploration of these genes and pathways may influence the development of novel therapies for the incurable disease [43]. Indeed, RA exerts neuroprotective functions, ensures the physiological structural integrity of the neurovascular unit in the brain, and may eradicate cancer stem cells in certain human cancers, including renal, lung, colorectal, and ovarian cancer [44,45,46]. Whether RA would deplete cancer stem cells from primary human brain tumors is less clear. In our previous work [7], we have shown that only a subpopulation of cancer stem cells from human GBM and GS specimens (referred to as SLGCs in the present manuscript) enter differentiation in response to RA and that the RA signal is transduced via RARα and RARγ in these cases [7]. Our previous data also suggested a contribution of RAR-independent RXR signaling to the treatment outcome, which we aimed to decipher in the present work.

IDH1-positive SLGC lines from six GBM and two GS specimens were investigated. Confirming our previous results, the effects of atRA varied between the SLGC lines. SLGCs from two GBMs and one GS showed increased proliferation rates after treatment with 1 µM atRA, and one GBM-derived (T1338) and one GS-derived (T1447) SLGC line displayed growth inhibition. The results for distinct biological and technical replicates, in particular T1338, T1440, and T1452, revealed substantial variations, which appeared related to the variations in growth behavior. Another complication is the fact that atRA may undergo isomerization into 9cRA in the medium [38], suggesting that the effects of atRA might have been mediated by RAR/RXR heterodimers or distinct RXR heterodimers, which are permissive to 9cRA signaling [21,22]. The RXR-selective agonist CD3254, which does not affect the activity of RAR isotypes [7], exerted a significant pro-proliferative effect in most SLGC lines and inhibited proliferation in two cases. Moreover, CD3254 did not significantly potentiate the growth inhibitory effects of the RAR pan-agonist CD0270 in most SLGC lines, suggesting that the pro-proliferative or pro-survival effects are exerted by RAR-independent pathways.

We isolated T1338 subclones that are homozygous for the p53 wildtype from the original heterogeneous T1338 culture and tested their growth behavior and atRA responsiveness. The growth behavior of these subclones differed, and most of them displayed a semi-adherent (s-adh) phenotype, i.e., they primarily formed adherent aggregates. When treated with 1 µM CD3254 (or bexarotene), the incorporation of BrdU was significantly augmented by both rexinoids, suggesting that rexinoids exert a pro-proliferative and/or pro-survival effect. Altogether, the RAR pan-agonist but not atRA mediated a significant reduction in the proliferation of the T1338 subclones. Substantial variations were observed between the biological and technical replicates. These are related to variations in growth behavior, which cannot be fully controlled, and the properties of the retinoids [7,38]. Major complications are that single cell suspensions of T1338 cells form adherent or floating aggregates within two days, that these aggregates fuse, and that single cells shuttle between the aggregates. Another complication is that the cells in the centers of aggregates will not receive the same doses of retinoids as the cells on the periphery. Treatment of distinct T1338 p53^WT/WT^ clones with RAR isotype-selective retinoids revealed that the atRA signal is transduced through RARα and RARγ. This is in keeping with the observation that the RARβ promoter is hypermethylated and associated with the repressive chromatin mark H3K9me2/3. In this context, it should be mentioned that histone lysine methyltransferase SETDB1, which mediates di-/tri-methylation of the histone H3 in the lysine residue K9, is thought to function as a common denominator of gene regulation in several cancer types, in which most data point to a pro-oncogenic role of SETDB1 [31].

The significance of the conclusions on RAR-dependent and RAR-independent RXR-signaling in SLGCs described above is limited by several parameters: first, several SLGCs form adherent and floating aggregates, which hampers the reproducibility of the experiments and the potency of retinoid treatment. Second, the use of the antibodies directed against the various RAR and RXR isotypes is limited by their K_D_s and specificities. Third, the RAR isotype-selective agonists are no longer selective when used at higher concentrations [7], and RXR isotype-selective ligands are not yet available. Fourth, though the SLGC lines are grown in a serum-free medium, it cannot be fully excluded that the medium component BIT (serum albumin-insulin-transferrin) contains traces of retinoids. Therefore, we decided to apply CRISPR/Cas9-mediated editing [33]. We chose a lentiviral vector [34] to overcome the low transfection efficacies associated with the SLGC phenotype. Despite the problems illustrated above, we selected the T1338 p53^WT/WT^ subclone T1338-1 for the editing approaches because of its high degree of stemness, the very high content of type I cells, and the good responsiveness to RAR- and RXR-selective ligands and TMZ.

The editing approaches were designed such that the sgRNAs targeted the exons of the RARA, RARB, RARG, RXRA, RXRB, and RXRG genes, which encode the first of the two zinc fingers. This strategy was chosen since the first zinc finger encompasses the so-called P-box, which mediates the recognition and binding of RARs and RXRs to their cognate response elements [20]. We used a lentiviral vector, which allowed for puromycin selection after the infection of the cells with the recombinant viruses. The efficacy of the puromycin selection, however, was hampered by the growth behavior of the T1338-1 cells. As could have been expected, a substantial number of wildtype clones were isolated from limited dilution experiments in addition to the edited clones. The editing of RARA and RXRA was highly efficient, as several isolated clones carried biallelic functional knockouts of the RARA and RXRG genes, respectively. The editing efficiency was considerably reduced for RARB and RARG, but also RXRB and RXRG. Moreover, most clones obtained from the RARB editing approach harbored RARB wildtype sequences. Since the wildtype sequences could not be removed by several additional rounds of limited dilution assays, this indicated that RARB editing occurred only in one allele in most of these clones. Considering the hypermethylation of the RARβ promoter and the increased proportion of the repressive mark H3K9me2/3, it could be speculated that the RARB gene was less accessible because of a comparatively closed chromatin state. Indeed, the efficacy of Cas9-mediated gene editing is dependent on chromatin accessibility [47]. Unexpectedly, the targeting of the RARA gene resulted in >90% of the cases having a homozygous insertion of a single nucleotide, which was an adenosine-monophosphate in 11 out of 12 cases and a thymidine-monophosphate in one single case. The editing approach of the RXRA gene was similarly efficient, with a slight preference for a homozygous insertion of a thymidine-monophosphate; but deletions of up to three nucleotides were also frequently observed. Biallelic editing of the RARG, RXRB, and RXRG genes was possible, but with a lower efficiency. The simultaneous editing of two RAR isotypes was possible, and signs of growth reduction did not become evident in the RAR double knockouts. In the case of the RXR editing approaches, only one clone (RXR H6αγ) with a full knockout of the RXRA and RXRG genes was obtained, and one other clone (RXR C5αγ) most likely harbored a biallelic knockout of the RXRA gene plus a monoallelic knockout of RXRG. In contrast to the clones with a functional knockout of only one RXR isotype, the clone RXR H6αγ displayed impaired growth. This was not observed for RXR C5αγ, which still carried one functional RXRG gene. Both clones, particularly RXR H6αγ, showed reduced expression of the LDHA. Considering that the RXR isotypes form heterodimers with several nuclear receptors, including the PPARs, which function as metabolic sensors and regulate carbohydrate and/or lipid metabolism [21,22], the reduced expression of LDHA could indicate that RXR isotypes are necessary for the metabolic adaptation of SLGCs. Notably, the expression of PMK2 was high in T1338 cells and remained unaffected by RXR editing.

The T1338 clones with functional inactivation of one RAR isotype or one RXR isotype as well as the clones RXR H6αγ and RXR C5αγ were selected for further experiments. The first central aim was the analysis of stemness. This was approached by double stains using antibodies against Sox2 and CD133. Except for one RAR (RAR F2γ) and two RXR clones (RXR C10α, RXR F4α), the cultures of the various RAR and RXR clones consisted primarily of Sox2+ cells that did not express CD133. Since the Sox2 signal was very strong in >95% of the cells, the respective cultures contained almost solely type I cells. Similar to the T1338 mother culture, all clones expressed the neuronal protein Tau and low levels of the glial fibrillary protein GFAP. In the cases of the RXR knockout clones, however, the expression levels of Tau and GFAP were increased, even in the presence of high levels of Sox2. This indicated that a high degree of stemness does not prevent the expression of Tau and GFAP and that the inactivation of one RXR isotype might result in higher levels of these neural proteins. Treatment with 1µM atRA/1 mM cAMP further increased the Tau and GFAP levels in the respective RXR clones and drastically decreased cell numbers. In the cases of the RAR clones, in which the effects of 1 µM atRA were assessed in the absence of cAMP, the treatment with 1 µM atRA reduced the cell numbers and moderately increased the expression of Tau and GFAP.

Experiments comparing the proliferation and growth of clones with edited RAR or RXR genes revealed substantial heterogeneity between clones with the same type of editing. Significant differences were also observed between the RAR and RXR mock clones, respectively. The direct comparison of the proliferation of two RXR (or RAR) mock clones with several edited RXR (or RAR) clones suggested that the homozygous inactivation of only one RXR (or RAR) isotype does not impair the ability of T1338 cells to stably proliferate in N-medium. Solely, the full inactivation of both, RXRα and RXRγ, which was achieved in the clone RXR H6αγ, impaired growth, probably reflecting the reduced availability of RXR isotypes for the formation of RXR permissive or non-permissive heterodimers [20,21,22]. In the presence of the RXR pan-agonist CD3254, a significant increase in proliferation was observed for both RXR mock clones. This pro-proliferative effect was reduced for the RXRA knockouts and absent or almost absent for the RXRB, RARG, and/or RXRA/RXRG knockouts. Hence, the knockout of one RXR isotype might be compensated by the two others, yet the limited numbers of RXR receptors might derogate the activity of several RXR-dependent nuclear receptor signaling pathways [20,21,22]. This could especially alleviate the capacity of T1338 (and presumably other SLGCs) to adapt to metabolic requirements [39,40]. The plausibility of such a scenario is revealed by reduced LDHA levels in the double knockout RXR H6αγ. In this context, it might also be interesting that RXRβ and RXRγ null mice are viable without abnormalities typically associated with vitamin A deficiency (VAD) or vitamin A excess, whereas the RXRα knockout resulted in severe fetal VAD syndrome and death (overview in [20]).

Several recent reports suggest a central role for RAR and RXR signaling during the specification of neural fates, neuronal differentiation, neurodegenerative disease, and brain cancer [19,20,21,22,48,49,50]. In order to evaluate whether RXR signaling might improve the cytotoxic effects of TMZ, we treated the RXR mock clones with the rexinoid bexarotene. We chose bexarotene for these assays since it has been approved by the FDA for clinical applications, it possesses more favorable K_D_s than CD3254, and the effects of bexarotene derivatives have been examined in the context of GBM cell lines [51]. Bexareotene did not deplete Sox2+ cells from the cultures of edited RXR clones or the mock controls, and 1 µM bexarotene mediated a dose-dependent pro-proliferative effect in a subset of clones. When co-applied with 25 µM TMZ, which exerted a strong cytotoxic effect, 1 µM bexarotene weakened the TMZ-mediated effect in a subset of clones. This effect was most pronounced in the clones with the RXRα/γ double knockouts and appeared otherwise independent of the type of RXR knockout. The phosphorylation of the histone variant H2AX (which generates γH2AX), which is used as a potential measure for the induction of DNA lesions [52,53], was detected in almost all cells that survived the five-day treatment with 25 µM TMZ. In contrast, γH2AX+ cells were nearly absent in the DMSO controls and the bexarotene-treated cultures of the edited RXR clones and the RXR mock controls. On average, the few cells that survived the five-day treatment with TMZ in the presence of bexarotene exhibited a similarly strong γH2AX signal as the survivors of the sole TMZ treatment. Finally, TMZ and bexarotene/TMZ mediated a similar strong reduction in Sox2-positive cells. Taken together, this suggests that the bexarotene signal may be transduced through any of the RXR isotypes. Moreover, bexarotene is unlikely to inhibit the growth of SLGCs or sensitize SLGCs for TMZ treatment. This is contradictory to two reports, which assessed the responsiveness of the rat glioma cell line C6 [54] or primary GBM cells [55] to bexarotene and postulated anti-proliferative effects. Since distinct driver mutations are present in SLGCs from distinct GBM (and GS) patients and SLGCS may exhibit adaptive plasticity [3,9], it cannot be excluded that bexarotene might sensitize a certain subgroup of SLGCs with specific features in their genomes and epigenomes.

## 4. Materials and Methods

Stem-like glioma cells (SLGCs), cell culture, and growth behavior: Experiments with cell lines derived from human tumor specimens were performed according to the Helsinki guidelines, in compliance with the national regulations for the experimental use of human material (vote 08-070 of the Ethics Commission at the University of Lübeck). SLGC (stem-like glioma cell) lines were established from human glioblastoma multiforme (GBM) or gliosarcoma (GS) [7,10,27]. Tumor specimens were obtained from patients who underwent routine tumor resection and expressed their agreement to participate in the study. The classification of tumor type and grade was according to the WHO criteria and carried out by a neuropathologist. Tumor specimens were made anonymous. The cell lines and subclones derived thereof were designated “T”, followed by the code number of the tumor plus a suffix in the case of clones. SLGCs were grown in serum-free N-medium, which contained DMEM/Ham’s F12 (Biochrom; Berlin, Germany), 20% BIT 9500 serum-free supplement (PELOBiotech GmbH, Planegg, Germany), 2% of a 200 mM glutamine solution, 1% amphotericin, 1% standard penicillin/streptomycin mix, and 20 ng/mL of recombinant human EGF and bFGF (Promo Cell, Heidelberg, Germany)). Passaging was performed with trypsin/EDTA (Invitrogen, Karlsruhe, Germany). All cell cultures were incubated at 37 °C in a water-saturated atmosphere in the presence of an air–carbon dioxide (5% CO_2_) mixture. Growth curves: Cells were seeded at a concentration of 1 × 10^4^ cells per well in 24-well plates (Greiner, Frickenhausen, Germany). These assays were performed in pure N-medium in the absence of fibronectin, to allow for spherical growth. Counting was performed on the days indicated in the figures; harvesting and dissociation of spheres were carried out using trypsin/EDTA, Trypan Blue (Sigma-Aldrich, Munich, Germany), and Neubauer counting chambers (VWR, Haasrode, Belgium). Assays were performed in triplicate and repeated with another biological replicate, which was regrown from a cryo-stock. Growth phenotype: Monitoring of the growth of spheroids, adherent aggregates, or monolayers was performed using the Incucyte^®^ SX (Sartorius, Göttingen, Germany). Cells were plated at 1 × 10^4^ cells per cm^2^ in N-medium and observed over 12 days.

Immunocytochemistry (ICC): Cells were analyzed on glass-coverslips or 8-well chamber slides. Adherence was increased by coating the growth substrates with fibronectin (1:100 dilution in 1× PBS; Promo Cell, Heidelberg, Germany). In order to prevent cell loss, all T1338 cells (subclones mother culture and edited clones) had to be analyzed in the presence of 2% fetal calf serum (FCS), which was added to the N-medium. N-medium containing 2% FCS will not induce differentiation [10]. Fixation was performed for 7 min at −20 °C using a mixture of ethanol–acetic acid (95:5 *v*:*v*). Primary and secondary antibodies are listed in the File S2 Section S6. DAPI nuclear counterstain was performed with Hoechst 33258 (Roche, Mannheim, Germany). Fluoromount-G (Southern Biotechnologies, Birmingham, USA) was used for mounting. Results were observed by fluorescence microscopy using the BZ8000 and the software BZ9000 (Keyence, Neu-Isenburg, Germany). Microphotographs of aggregates and spheres were taken as Z-stacks (pitch 0.2 to 0.5). A minimum of three independent biological replicates, including two technical replicates each, were analyzed. The quantification of the signal intensities was performed using the software QuPath (version v0.2.3). Based on the model of cellular hierarchy [17], CD133-negative cells with high Sox2 expression are classified as type I cells; type II cells co-express Sox2 and CD133; CD133-negative cells with lower Sox2 expression are classified as type III cells. DAPI nuclear counterstain was omitted from the images prior to quantification.

Western blot analysis: Cells were harvested in 1 mL of TEN [10 mM Tris-HCl (pH 7.5), 1 mM EDTA (pH 8.0), and 150 mM NaCl]. Protein extraction was performed with a buffer containing 50 mM Tris-HCl (pH7.5), 150 mM NaCl, 10% glycerol (*v*:*v*), 0.5% Triton-X100 (*v*:*v*), 0.1% of a saturated PMSF solution (Phenylmethylsulfonylfluorid; Sigma-Aldrich, Munich, Germany), and a protease inhibitor cocktail (PIC; Roche, Mannheim, Germany). Cell debris was removed by a high-speed clearing spin. In total, 15 µg (p21^CIP1^, Sox2, PKM2, LDHA), and 20 µg (MGMT, RAR and RXR antibodies) of whole cell extracts (WCE) were resolved by SDS-PAGE (Minigel Protean III, BioRad, Munich, Germany) and transferred onto nitrocellulose membranes (0.45 µm, BioRad) using the Semidry Transfer System TransBlot SD (BioRad). Electrophoresis was carried out with run buffer (25 mM Trizma Base, 192 mM glycine, 0.1% SDS); a buffer containing 48 mM Trizma Base (pH 8.3), 39 mM glycine, 0.037% SDS (*w*:*v*), and 20% methanol (*v*:*v*) was used for protein transfer. Separation of WCEs was performed on 15% resolving gels for MGMT and p21^CIP^; in all other cases, resolving gels contained 10% polyacrylamide and stacking gels of 4%. The loading controls used to normalize protein expression were pan-Actin and GAPDH (glyceraldehyde 3-phosphate dehydrogenase). Primary antibodies (see File S2 Section S6) were revealed using horseradish peroxidase- (POD-) conjugated secondary antibodies (see, File S2 Section S6) and Super Signal^®^ West Dura Extended Duration Substrate (#34076; Thermo Fisher Scientific; Loughborough, UK) or Clarity Max Western ECL Substrate (#1705062, BioRad). Chemiluminescence was detected with the ChemiDoc XRS system (BioRad), and quantification was performed with the software Quantity One versions 4.6.2-4.6.6 (BioRad). Western blots included a minimum of three biological replicates, two of which were regrown from cryostocks. Depending on how much WCE was available from the individual experiments, 2–4 technical replicates were produced with the same WCE.

Methylation-specific PCR (MSP): Genomic DNA was extracted using the DNeasy Mini-Kit (#69504, Qiagen, Hilden, Germany) followed by quantitative and qualitative characterization using standard protocols. Bisulfite modification of 1 µg of DNA and subsequent purification on glass milk were performed with the CpGenome^TM^ DNA Modification Kit reagents (#S7820; Merck Millipore, Darmstadt, Germany) according to the manufacturer’s instructions. PCR was performed using standard protocols [56] (for primers, see File S2 Section S7). Nucleotides (dATP, dCTP, dGTP, dTTP, 10 mM each) were purchased from Roche (Mannheim, Germany, #04638956001), and Taq polymerase (5 units/µL plus 10× buffer) from VWR International (Haasrode, Belgium; #VWR-733-1300). PCR products were mixed with 6× orange loading dye (Thermo Fisher Scientific, Schwerte, Germany), run on 2 % TBE agarose, and quantified using the ChemiDoc XRS and the software *Quantity One* (BioRad, Munich, Germany). The MSP of treated cells included a minimum of three independent technical replicates. The MGMT status of the SLGC lines was determined in >5 biological replicates.

Genomic editing using the CRISPR-Cas9 system [33]: For single guide (sg) RNA design and exclusion of OFF targets, we applied several online tools (http://www.rgenome.net/), including CRISPR RGEN, Cas-OFFinder, Cas-Designer, BE-Analyzer—RGEN, and the ENSEMBL Genome Browser (https://www.ensembl.org/index.html). Cloning of the DNA coding for the sgRNA was performed using standard protocols [56,57]. In brief, the vector lenti-CRISPR v2 (Plasmid #52961; Addgene Cambridge, MA, USA [34]) was cleaved with the enzyme BsmBI (#R0580S, New England Biolabs, Ipswich, MA, USA), dephosphorylated using calf intestinal alkaline phosphatase (CIAP, #18009019, Thermo Fisher Scientic), and purified by phenol/chloroform extraction and ethanol precipitation. The oligonucleotides for the sgDNA cloning were phosphorylated using den T4 PNK (#MO201S; New England Biolabs), followed by the inactivation of the enzyme. Afterward, the oligonucleotides were annealed at a concentration of 10 µM each. The annealing product was inserted into the lenti-CRISPR v2 vector using the T4 Quick ligase (#M2200S, New England Biolabs), followed by transfection into *E. coli* K12 Stbl3 (#C737303, Thermo Fisher Scientific). Plasmid DNA was extracted by applying a standard alkaline lysis protocol, including phenol/chloroform extraction and ethanol precipitation [56,57]; correct insertion of the sgDNA was determined by means of Sanger sequencing using the BrilliantDye™ Terminator (v3.1) Cycle Sequencing Kit (100 rxn, #BRD3-100, NimaGen, Nijmegen, The Netherlands). After purification on NucleoSEQ^®^ columns (#740523.250, Macherey-Nagel, Düren, Germany) and denaturation in the presence of HiDi^TM^ Formamide (Thermo Fisher Scientic), the samples were analyzed on the Applied Biosystems 3130 Genetic Analyzer using the Sequencing Analysis Software v5.2 (Applied Biosystems, Foster City, USA). Sequence analyses were carried out by means of Chromas (Technelysium, South Brisbane, AUS) and the ENSEMBL Genome Browser (https://www.ensembl.org/index.html). The recombinant lenti-CRISPR v2 vectors were co-transfected with two packaging plasmids (psPAX2 (Addgene plasmid #12260) and pCMV-VSV-G (Addgene plasmid #8454)) into HEK293T cells (American Type Culture, Manassas, VA, USA) using the calcium-phosphate technique. Virus preparations were purified through 0.45 µm PES Millex ^®^ filters (Merck Millipore). Infection of the target cell line T1338-1 (referred to as T1338 MC afterward) was in the presence of Polypren (8 mg/mL, Sigma-Aldrich, 1:1,000 dilution). Selection with Puromycin (Life Technologies^TM^, Carlsbad, CA, USA) was carried out at a concentration of 1 µg/mL for seven days. Limited dilution assays were used to isolate clones, which were subsequently expanded to obtain enough material for cryo-conservation and gDNA extraction (DNeasy, #69504, Qiagen, Hilden, Germany). Genomic editing was revealed by PCR-mediated amplification of the targeted exon using the respective RAR or RXR isotype-selective primer pairs, followed by purification on TBE-agarose gels and spun column chromatography (NucleoSpin Gel and PCR Clean-Up Kit, #740609.50, Macherey-Nagel). Subsequent Sanger sequencing was performed as described above, using purified PCR products or recombinant pCR^TM^2.1-TOPO^®^ DNA; amino acid sequences were deduced using the Expasy tool that was developed by the SIB (Swiss Institute of Bioinformatics, Lausanne, Switzerland). Recombinant plasmids were generated by TA cloning (Eukaryotic TA Cloning Kit, #2474228, Invitrogen) of the purified PCR fragments into the vector pCR^TM^2.1-TOPO^®^ (Invitrogen). For amplification of recombinant pCR^TM^2.1-TOPO^®^, we used E.coli K12 DH5α (initially obtained from Invitrogen); plasmid extraction was done by alkaline lysis [56,57]; for sequencing of pCR^TM^2.1-TOPO^®^, we used the primers M13-62-ex_F and M13-62-ex_R, respectively. In those cases in which the sequencing data suggested a heterogeneous cell population and/or the presence of residual wildtype sequences, additional rounds of limited dilution assays were carried out. This was necessary for the isolation of clones with edited RARβ, RARγ, RXRβ, and RXRγ genes. The editing of the RARβ gene was particularly ineffective. In spite of three additional rounds of limited dilution assays, three RARβ clones still contained RARβ wildtype sequences, indicating that biallelic editing did not occur. The primers used for editing, cloning, or sequencing are listed in the File S2 Section S7.

Synthetic retinoids and additional reagents: Temozolomide (TMZ, Temodal^®^; Essex Pharma GmbH, Munich, Germany); all-trans retinoic acid (atRA, Sigma-Aldrich, Munich, Germany); pan RAR agonist: CD0270 (Galderma Laboratories, Düsseldorf, Germany); RXR pan agonists: CD3234 (K_D_ 50 nM for RXRα, RXRβ, and RXRγ; Tocris Bioscience), Bexarotene (K_D_ 24 nM RXRβ, 25 nM RXRγ, 33 nM RXRα; Tocris Bioscience); RARα agonists: CD1556 (Galderma Laboratories); Am580 (EC50 0.3 nM RAR, 8.6 nM RARβ, 13 nM RARγ; Tocris Bioscience, Minneapolis, MN, USA); RARβ agonist: CD1886 (Galderma Laboratories); RARγ agonist: CD2640 (Galderma Laboratories). The K_D_s of the synthetic retinoids CD0270, CD1556, CD1886, and CD2640 have already been described in our previous study [7]. All compounds were dissolved in DMSO. Notably, all assays without TMZ and/or ligands contained a final concentration of 1% DMSO.

BrdU-ELISA: These assays were performed on fibronectin-coated 96-well plates. Each experiment included a minimum of two biological replicates. Two identical plates, each encompassing eight independent values, were analyzed. The responsiveness of distinct SLGC lines was monitored on days d3, d5, and d7. For the T1338 line, which was selected for further experiments, more than 15 biological replicates (subclones; T1338-1 is referred to as MC, mother culture) were analyzed. For the clones with RAR or RXR edits, a minimum of three biological replicates were analyzed. In order to prevent cell loss, all assays with T1338 cells had to be performed in the presence of 2% fetal calf serum (FCS), which does not impact the degree of stemness in the presence of the growth factors EGF and bFGF [10]. Coating with fibronectin (5 µg/mL; Promo Cell, Heidelberg, Germany) was at a dilution of 1:100. Analyses were initially performed using the kits marketed by Roche (Mannheim, Germany; #114446141001 or #11647229001) and continued using the BrdU Cell Proliferation Assay Kit marketed by Cell Signaling (Danvers, MA, USA, #6813S). Due to the suspension of deliveries, another BrdU kit (BrdU Cell Proliferation Assay Kit, #6813, Cell Signaling) had to be implemented into the study at later stages. All experiments were carried out according to the manufacturer’s instructions.

Chromatin immunoprecipitation (ChIP): Cells were analyzed by ChIP during their exponential growth phase. Floating spheres were harvested by centrifugation (10 min, 250× *g*, 4 °C), and semi-adherent and adherent cells were detached with a rubber policeman. We used the simpleChIP^TM^ enzymatic chromatin IP Kit (Cell Signaling, Danvers, MA, USA), applying the manufacturer’s protocol with several adaptations. These concerned the treatment with the micrococcal nuclease (duration, concentration of enzyme), the sonification steps (pulses and number of sonification cycles), and the immunoprecipitation steps, which had to be optimized for each individual cell line and antibody. Reversal of the cross-link was achieved with proteinase K followed by spin column purification (kit components). Only chromatin preparations with mono- to penta-nucleosomes were used. Immunoprecipitation was preceded by a preclearing step (2–4 h on a rotator at 4 °C) using a mixture of Protein A and Protein G agarose beads (Cell Signaling). The chromatin was incubated for 4 h with the antibodies before capture was carried out with Protein G agarose beads (4 °C, overnight, on a rotator). The washing steps and release of the immunoprecipitated DNA were performed according to the simpleChIP^TM^ enzymatic chromatin IP Kit protocol. All assays included precipitations with a suitable, unrelated IgG antibody. At least three independent PCR reactions were carried out. The number of amplification cycles was 28–32 for *rpl*30 and 30–35 for *rarβ*. PCR products were resolved on 1.6 % TBE-agarose gels and quantified using the ChemiDoc XRS and the Quantity One” software (BioRad). The primer pair *rpl30* F/R was obtained from Cell Signaling (simpleChIP^TM^ Kit); all other primers used for ChIP are listed in the File S2 Section S7. The antibodies used were αIgG (#2729, Cell Signaling), αH3 (#2650, Cell Signaling), αH3K9me2/3 (#5327, Cell Signaling), and αH3K9ac (#9674, Cell Signaling).

qRT-PCR analysis: Extraction of total RNA was performed using the RNeasy Mini-Kit (Qiagen) followed by a DNase I-treatment (Ambion, Darmstadt, Germany), and extraction with phenol-chloroform (Roth) and ethanol (Merck) precipitation was according to standard protocols [56]. cDNA synthesis was performed with the iScript^TM^ Reverse Transcription Supermix for RT-qPCR kit (BioRad, Munich, Germany) followed by second strand synthesis with the qPCR Core kit for SYBR^®^ Green I kit (Eurogentec, Cologne, Germany) and monitoring of product synthesis with the CFX96 Real-time PCR Detection System (BioRad). Quantification of the PCR products was relative to the reference genes *gapdh*, *ubiquitin ligase*, and 18s *r-rna*. Relative expression was calculated according to the ΔΔ-CT method. Analyses were performed in triplicate, using RNA from two biological replicates. DNA-oligonucleotides were purchased from Eurofins genomics (Ebersberg, Germany). The respective sequences have been published elsewhere [7].

Statistical analysis was performed using GraphPad Prism version 9 (Boston, MA, USA). In order to determine significant differences in BrdU ELISA, growth curves, Western blots, immunocytochemistry analyses, and qRT-PCR analyses, a one-sided ANOVA followed by post hoc Bonferroni correction for multiple testing was used; adjusted *p*-values are indicated in the figures and legends to the figures. Since the K_D_s of the antibodies used for ChIP are not known and since the efficacy of bisulfate modification may vary, descriptive statistics were used for ChIP and MSP; mean values and standard deviations are indicated in the respective figures.

## 5. Conclusions

The present analysis of six GBM- and two GS-derived SLGC lines, all of which are IDH1-wildtype but differ in their p53 and MGMT status, suggests that the activation of the RAR/RXR heterodimer may reduce proliferation in several but not all SLGC lines. Inhibition of proliferation shifts a small proportion of the type I cells into the progenitor states (type II and III cells) but does not lead to terminal differentiation. RAR-independent RXR signaling has the potential to augment proliferation in certain SLGC lines.

Functional knockouts of the three RAR or RXR isotypes in the IDH1/p53 wildtype T1338-1 subclone, which does not express the detoxifying enzyme MGMT, indicated that stemness, proliferation, and viability of T1338-1 cells are not affected by CRISPR/Cas9-mediated editing of the corresponding genes. Also, T1338-1 clones with a knockout of two RAR isotypes generated stably proliferating clones, whereas the double knockout of RXR*α*/RXRγ impaired growth. Moreover, RXR signaling may affect the capacity of SLGCs to adjust their metabolic pathways to changes in the microenvironment, as the expression of the lactate dehydrogenase LDHA was reduced in RXR*α*/γ double knockout.

Our results support the conclusion that the responsiveness to TMZ cannot be improved by stimulation of RAR-independent RXR signaling pathways. Rather, the present work fosters the presumption that RXR-selective ligands might exert a pro-proliferative and/or pro-survival effect and could weaken the cytotoxic effects of TMZ.

## Data Availability

All data and materials are available from the corresponding author on request.

## Supplementary Materials

The following supporting information can be downloaded at: https://www.mdpi.com/article/10.3390/ijms242216466/s1.
