# Supplementary material for: RAR-Dependent and RAR-Independent RXR Signaling in Stem-like Glioma Cells
Source: Int J Mol Sci. 2023 Nov 17;24(22):16466. doi: 10.3390/ijms242216466 (PMC10671216; doi:10.3390/ijms242216466)
Supplement: Supplementary file 1 [file ijms-24-16466-s001.zip › File S2_supplementary information.pdf]

File S2 – Supplementary information

S1: Concept of the study and limitations associated with growth behavior:
Concept: First, we investigated the responsiveness of eight distinct SLGC lines to the treatment with a rexinoid. We measured proliferation and determined the impact on stemness and differentiation. This led to the selection of the T1338 line for further experiments. However, this required the selection of a suitable sublone. Having chosen a subclone, it was important to test whether the clone would express all three RAR and RXR isotypes, for which the expression analyses of RARβ were demanding since western blot and qRT-PCR data were contradictory. Moreover, the status of the RARβ promoter was important for understanding the outcome of the editing experiments. We targeted all three RAR and RXR isotype genes. One major parameter was the potential impact of the respective knockout on proliferation, stemness and differentiation capacity. In the case of the RXR knockout clones, we further extended the study and analyzed whether the knockouts would impact glycolytic enzymes and the responsiveness to TMZ.
Limitations: The present study investigates the behavior of stem-like glioma cells, which tend to form spheroids when grown in serum-free medium. The degree of spheroidal growth, however, varies between SLGC lines from distinct tumor specimens and between biological replicates of the same SLGC lines. In most cases, the spheroids tend to form adherent aggregates on the growth substrates when kept in the culture dish for two or more days. Again, this process is highly variable and involves the integration and release of single cells from the respective aggregates. We could monitor this process in detail in 2018, when he had the option to test the Incucyte System. The respective data were entered into a manuscript that is under review; however, a few aspects of this investigation are shown in Fig. 1B. What is even more important than the variations in growth behavior is the limited access of the reagents used in the treatment to cells in the centers of the aggregates and the metabolic changes that occur in these aggregates during the course of the experiments.

S2: Degree of Stemness: GBM and GS cells with features of neural stem cells express the transcription factor Sox2 [15]. When the cells start to differentiate, Sox2 becomes downregulated and the glycoprotein CD133 is transiently upregulated [17]. Three distinct differentiation states of stem-like glioma cells were defined by Chen et al. [17]: (i) the Sox2+/CD133- type I cell (glioma stem cell); (ii) Sox2+/CD133+ type II cell (progenitor); (iii) Sox2(+)/CD133- type III cell (late progenitor). Changes in the relative numbers of type I, II and III cells constitute, therefore, a useful tool for the monitoring of differentiation.
We observe the presence of type I, II, and III cells by immunocytochemistry analyses using antibodies against Sox2 and CD133. Two distinct combinations of antibodies were used for this purpose: the first combination uses a mouse-anti-Sox2 antibody together with a rabbit-anti-CD133 antibody. The second combination combined a mouse-anti-CD133 antibody with a rabbit-anti-Sox2 antibody. Both antibody combinations revealed similar but not identical expression patterns of the respective proteins. In particular, the binding of the transcription factor Sox2 to defined chromatin territories is more evident in experiments using the mouse-Sox2-antibody (examples in Fig. 1D). The quantification of Sox2 expression levels, however, is more feasible with the rabbit-Sox2 antibody. In this context, it has to be noted that the rabbit-CD133 antibody is no longer available on the market.

S3: Induction and Monitoring of Differentiation:
To evaluate the capacity of atRA, and the RAR- or RXR-selective pan agonists for the induction of neural differentiation, immunocytochemistry analyses were performed. The degree of stemness was deduced from the expression of the transcription factor Sox2 and the glycoprotein CD133 [15-17], the induction of neural differentiation by the expression of the glial fibrillary protein GFAP [27] and the microtubule-associated protein Tau [28]. In this context, it has to be noted that GFAP is absent in most but not all undifferentiated SLGC lines [7,10]. Notably, high levels of GFAP and Sox2 are co-expressed in a subgroup of our SLGCs (e.g., T1440 and T1452) and GFAP becomes downregulated when these SLGCs undergo differentiation [7,10]. On the contrary, the SLGC lines encompassing GFAP-/Sox2+ type I cells

induce GFAP during differentiation [7]. The protein Tau is expressed in all SLGC lines, and Tau levels significantly increase after the induction of differentiation [7].

S4: Necessity for the Selection of a T1338 Subclone Suitable for Further Experiments:

During the course of the experiments, we noticed that the effects of atRA, CD0270, and CD3254 on proliferation were more evident for some biological replicates of T1338 cells than others (see, e.g., the variation of optical densities in Fig. 1D). Moreover, we recognized that the original T1338 culture encompassed two subpopulations of cells, one of which carried the wildtype tumor suppressor p53 ($p53^{WT/WT}$), whereas the other one displayed a $p53^{WT/mut}$ status [27]. To exclude the possibility that the heterogeneity in the responsiveness could be due to the relative abundance of $p53^{WT/mut}$ and $p53^{WT/WT}$ cells, we applied limited assays to isolate T1338 cells with a $p53^{WT/WT}$ status. Seven T1338 $p53^{WT/WT}$ clones were isolated. The respective clones were characterized, and their responsiveness to retinoids was investigated.

All T1338 $p53^{WT/WT}$ clone cultures exhibited a high expression of Sox2 in >95% of the cells but showed variations in growth behavior (examples in Figure S3A). Whereas some clones primarily formed monolayers plus adherent aggregates (*adh*), others primarily encompassed adherent aggregates (*s-adh*) of distinct size, and a third group primarily consisted of spheroids and adherent aggregates (*sph*). Two subclones with an *adh* phenotype (T1338-5, -6), four clones with an *s-adh* (T1338-1, -2, -3, -11) and one with an *s-adh/sph* (T1338-9) phenotype were selected for further analyses. In this context, it has to be noted that growth behavior and the size of aggregates and spheroids vary within certain ranges (Figure S3A). When these T1338 subclones were analyzed for their responsiveness to 1 μM atRA, 1 μM of a pan RAR agonist, or 1 μM of the rexinoids CD3254 and bexarotene, the mean values of the respective BrdU ELISA showed a substantial variation in all cases (examples in Figure 2A). Yet, the rexinoid mediated an increase in proliferation, whereas the RAR agonist CD0270 impaired BrdU incorporation, confirming the data obtained for the T1338 mother culture (Figures 1B, 2A). Since the efficacy of ligand treatment decreased for T1338 clones with huge adherent or floating aggregates or a pronounced *sph* phenotype, T1338-1 was selected for further experiments. Moreover, T1338-1 showed good responsiveness to TMZ (Figure 2A). This is essential since one basic aim of the present study concerns the analysis whether synthetic RAR- or RXR-selective ligands might improve standard GBM therapy.

Several distinct antibodies were used in order to reveal the presence of RAR and RXR protein expression (see Section Materials and Methods). Unfortunately, unambiguous and reproducible Western blot data were only obtained with the antibodies directed against RARβ or RXRβ, whereas all other antibodies generated flimsy (anti-RARα, -RARγ, or -RXRα) or no signals (anti-RXRγ). The respective Western blot analyses (examples in figure S3C) suggested high levels of RARβ and RXRβ proteins in T1338 cells, which remained largely unchanged after treatment with atRA or synthetic retinoids. Quantitative RT-PCR analyses revealed the presence of RARα, RARβ, and RARγ mRNAs, in which the levels of RARα and RARγ mRNAs were 10-fold higher than those of the RARβ mRNA (Figure S3D). RXR–α, RXR,–β and RXR–γ specific antibodies revealed the presence of the respective receptors in the nucleus of Sox2-positive T1338-1 cells (examples in figure S3D). The signals, however, were too weak to deduce any treatment-induced changes in the expression levels (data not shown). To summarize, all three RAR and RXR isotypes are expressed in T1338-1 and could contribute to the transduction of the RA signal.

Since the RARβ isotype, which deserves specific attention [18,24], appeared highly expressed according to the Western blot data but displayed the lowest expression in qRT-PCR analyses (Figs. S3C, S3D), it appeared important to clarify these contradictory results through additional experiments. This was achieved by means of methylation-specific PCR (MSP) and chromatin immunoprecipitation (ChIP). MSP analyses (examples in Figs. 2B, 2C) using four distinct biological replicates of the T1338-1 subclone indicated a high degree of methylation of the RARβ gene promoter, which remained largely unchanged after treatment with atRA, RAR-selective agonists, the rexinoid CD3254 or combinations of RAR-selective agonists and CD3254 (Fig. 2C). In contrast, methylation of the RARα gene promoter became reduced after co-stimulation of RAR and RXR signaling (Fig. 2C). In keeping with the immunocytochemistry analyses investigating the stemness state, the Sox2 gene promoter was hypomethylated in the T1338-1 subclone and atRA and the synthetic retinoids induced only minor changes (Fig. 2C).

For the ChIP assays, we selected antibodies directed against modifications in the lysine residue 9 of histone H3 (H3K9). This decision was based on publications indicating that di- and tri-methylation of H3K9 by several SET-domain methyltransferases might be crucial in the development and treatment of several types of cancer, including malignant brain tumors [30-32]. In the course of these experiments, we compared the T1338-1 subclone to other T1338 subclones, one with an *s-adh* and one with an *adh* phenotype. The immunoprecipitations using chromatin from the T1338 subclones, and the antibodies against H3K9ac (a histone mark indicating activation) and H3K9me2/3 (a histone mark indicating repression) revealed a certain heterogeneity of the H3K9 status in the RARβ promoter. The two T1338 subclones with the s-adh phenotype (including T1338-1) displayed similar degrees of H3K9ac and H3K9me2/3, whereas the T1338-adh clone exhibited higher levels of H3K9ac (examples in Fig. 2D). A reduction of H3K9 methylation by atRA or atRA/cAMP was not evident (Fig. 2D, middle). This is in contrast to the RLP30 promoter, which is active in all cells, the chromatin of the RARβ gene promoter appeared more closed (Fig. 2D). In this context, it has to be noted that all antibodies used for ChIP were assigned for this purpose by the manufacturer but may exhibit distinct K_Ds, which may limit the validity of the assays.

S5: RAR double knock outs: In a second round of editing, we tested whether a knockout of two distinct RAR isotype genes would be possible in the same cell (double knockout). This was performed with the same T1338-1 mother culture (Fig. 3E), since we wanted to exclude the presence of any modifications that might have occurred in the genome or epigenome during a previous round of editing. As before, the editing of the RARα gene occurred with high efficacy, and resulted in the homozygous insertion of mostly an additional adenosine monophosphate. The editing of the RARβ and RARγ genes was similarly inefficient and heterogeneous as in the cases of the single knockouts (Figures S4, S5). Though this data indicates that all types of double knockout were possible, we did not further purify or analyze the respective clones. The reasons for this were the reduced viability of single T1338-1 cells under limited dilution conditions, the length of time from starting the limited dilution assay till the selection of clones (3-4 months), and the high probability of mono-allelic RARβ and RARγ knockouts. It should be noted in this context that Sanger sequencing verified that erroneous editing of the RARα genes was not observed when sgRNAs targeting RARβ and RARγ were used. The same degree of specificity was obtained when RARα and RARγ or RARα and RARβ were simultaneously targeted.

S6: ***Antibodies used for Western Blots or ICC***
Primary antibodies: pan-Actin (mouse; #MAB1504, Merck Millipore; Darmstadt, Germany); CD133/Prominin-1 (mouse, W6B3C1; #130-092-395, Miltenyi Biotec, Bergisch Gladbach, Germany; rabbit, clone C24B9, Cell Signaling, Danvers, MA, USA); GFAP (mouse, #MAB360, Merck Millipore; rabbit, #AB5804, Merck Millipore); GAPDH-POD (rabbit, peroxidase-coupled Ab #3683, Cell Signaling); Glycolysis Antibody Sampler Kit (includes anti-LDHA antibody; # 8337; Cell Signaling); γH2AX (rabbit; #9718, Cell Signaling); Nestin (mouse anti-human; #MAB5326, Merck Millipore); MGMT (mouse; clMT3.1; #MAB16200, Merck Millipore); p21^{CIP1} (mouse; DCS60; #2946, Cell Signaling); PKM2 (rabbit; D78A4; #4053, Cell Signaling); RAR and RXR antibody sampler kit (rabbit RARα (E6Z6K); rabbit RARγ1 (D3A4) XP®; rabbit RXRα (D6H10), rabbit RXRβ, rabbit RXRγ, #8589, Cell Signaling); RARα (2C9-1F8; #117728, Abcam); RARβ (#abEPR2017/AB124701, Abcam); RXRα (mouse, K8508; #433900, Thermo Fisher); RXRβ (rabbit, #AB24363; Abcam); RXRγ (rabbit, #PA5-110337; Thermo Fisher Scientific, Schwerte, Germany); Sox2 (rabbit; clone D6D9; #3579, Cell Signaling; mouse, 57CT 23.3.4; #79351, Abcam); Tau (rabbit, #6402, Sigma-Aldrich, Munich, Germany); alpha-Tubulin-POD (rabbit, 11H10; peroxidase-coupled Ab #9099, Cell Signaling).
Secondary antibodies: goat anti-mouse-POD (Goat F(ab')2 Fragment Anti-Mouse IgG (H+L) Peroxidase, #I-IMO817, Beckman Coulter, Krefeld, Germany); goat anti-rabbit-POD (Goat F(ab')2 Fragment Anti-rabbit IgG (H+L) Peroxidase; #I-IMO831, Beckman Coulter), goat anti-rabbit HRP (Cell Signaling), goat anti-mouse DyLight® (#96871, Abcam); goat anti-rabbit Cy3 (#111-165-003, Jackson Immuno-Research, Newmarket, Suffolk, UK).

S7: ***Oligo nucleotides***: Primers were purchased from Eurofins Genomics (Ebersberg, Germany)**.**

Primers for MSP: RARα^{meth} (F: 5′-GGT TTC GCG CGA TTC GGT TTT AC-3′; R: 5′-CTC TCG CTA AAG TCG CCT AAC GAC G-3′); RARα^{unmeth} (F: 5′-GGT TTT GTG TGA TTT GGT TTT AT-3′; R: 5′-CTC TCA CTA AAC TCA CCT AAC AAT A-3′) ; RARβ^{meth} (F: 5′-TCG AGA ACG CGA GCG ATT CG-3′; R: 5′-GAC CAA TCC AAC CGA AAC GA-3′) RARβ^{unmeth} (F: 5′-TTG AGA ATG TGA GTG ATT TGA-3′ ; R: 5′-AAC CAA TCC AAC CAA AAC AA-3′); Sox2meth (F: 5′-TGT TTA TTT ATT TTT TTC GAA AAG GCG G-3′; R: 5′-GAA CCC AAC CTC GCT ACC GAA-3′); Sox2^{unmeth} (F: 5′-TGT TTA TTT ATT TTT TTT GAA AAG GTG-3′; R: 5′-CTC AAA CCC AAC CTC ACT ACC AA-3′); MGMT^{meth} (M-F: 5′-GTT TTT AGA ACG TTT TGC GTT TCG AC -3′; M-R: 5′-5′-CAC CGT CCC GAA AAA AAA CTC CG-3′-3′); MGMT^{unmeth} (U-F: 5′-TGT GTT TTT AGA ATG TTT TGT GTT TTG AT-3′-3′; U-R: 5′-CTA CCA CCA TCC CAA AAA AAA ACT CCA -3′). ***Primers for ChIP***: RARB: (F: 5′-GCC GAG AAC GCG AGC GAT CC-3′; R: 5′-GGC CAA TCC AGC CGG GGC-3′); RPL30: kit component/sequence not known.

Primers for PCR on genomic DNA and direct sequencing of DBDs: RARA-exon 3 (F: 5′- AGA GGC TCT TAG GAG GGA CG -3′; R: 5′- GTG GGG ACA TCC CAT TGA CC -3′); RARB-exon 2 (5′- AGA AGC ATA TGA ATT CAC TGT TG -3′; R: 5′- GTA GGT CAC CTC CAT AAA GTA C -3′); RARG-exon 4 (5′-TGT GTG ATG AGG AAG CCT GG -3′; R: 5′- TGT GTG CCT GGT CTC TCA TC -3′); RXRA-exon 4 (F: 5′-GTA GTG GCG GCG TTG GAT G -3′; R: 5′-GGT GTC CAC GCA CAA GCC-3′); RXRB-exon 4 (F: 5′-GGT GAA GGT GTC TCC ATG C-3′; R: 5′- GTG GGA TAA GGG AGA AGG G-3′), RXRG-exon 4 (F: 5′-GCT TTC TGT CCC AAT GCC C –3′; R: 5′- CCC TTG CCT AGG AAA TGC C-3′).

Primers for plasmid sequencing: (i) pCR™2.1-TOPO® vector (M13-62-ex_F: 5′-GTT GTA AAA CGA CGG CCA GTG-3′;M13-62-ex_R: 5′- TCA CAC AGG AAA CAG CTA TGA C-3′); (ii) lenti-CRISPR V2-vector (lenti-F1: 5′-GAC TAT CAT ATG CTT ACC GTA AC-3′; lenti-R1: 5′-TCA AGT TGA TAA CGG ACT AGC C-3′).

Primers for cloning of single guide RNAs: RARA (F: 5′-CAC CGA AGC AAG GCT TGT AGA TGC G-3′; R: 5′-AAA CCG CAT CTA CAA GCC TTG CTT C-3′); RARB (F: 5′-CAC CGG TTT GTA CAC TCG AGG GGG-3′; R: 5′-AAA CCC CCC TCG AGT GTA CAA ACC-3′); RARG (F: 5′-CAC CGA AGC ATG GCT TGT AGA CCC G-3′; R: 5′-AAA CCG GGT CTA CAA GCC ATG CTT C-3′); RXRA (F: 5′-CAC CGC TAT GGA GTG TAC AGC TGC G-3′; R: 5′-AAA CCG CAG CTG TAC ACT CCA TAG C-3′); RXRB (F: 5′-CAC CGT TAC AGC TGT GAG GGT TGC A-3′; R: 5′-AAA CTG CAA CCC TCA CAG CTG TAA C-3′); RXRG (F: 5′-CAC CGT ACG GGG TAT ACA GTT GTG A-3′; R: 5′-AAA CTC ACA ACT GTA TAC CCC GTA C-3′).
