# Supplementary material for: RAR-Dependent and RAR-Independent RXR Signaling in Stem-like Glioma Cells
Source: Int J Mol Sci. 2023 Nov 17;24(22):16466. doi: 10.3390/ijms242216466 (PMC10671216; doi:10.3390/ijms242216466)
Supplement: Supplementary file 1 [file ijms-24-16466-s001.zip › File_S1_supplementary figures & legends.pdf]

Supplementary File S1_ supplementary figures

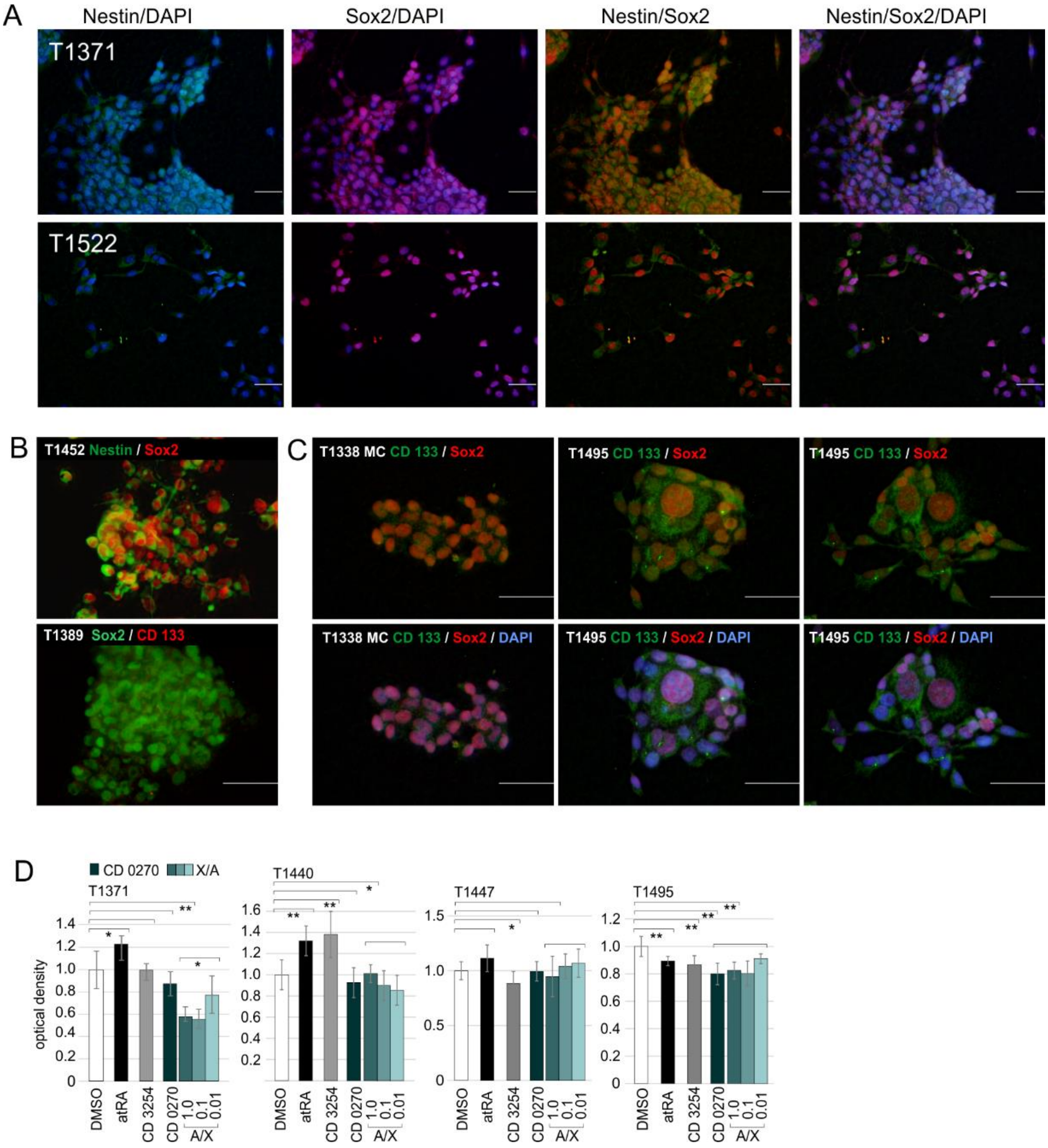

Figure S1. Characteristics of SLGC lines and responsiveness to atRA, CD0270 and/or CD3254. **(A)** Immunocytochemistry analyses: comparison of the stemness states of the SLGC lines T1371 and T1522. The antibody combinations mouse-anti-CD133/goat-anti-mouse DyLight© and rabbit-anti-Sox2/goat-anti-rabbit Cy3 were used. Nuclear counterstain was performed with DAPI (4',6-Diamidino-2-phenylindol)). **(B)** A similar experiment as in (A) using a mouse-anti-Sox2 and a rabbit-anti-Sox2 antibody, respectively. (**C**) A similar experiment as in (A) using the antibody pairs mouse-anti-CD133 and rabbit-anti-Sox2. The microphotographs with and without DAPI nuclear counterstain are depicted on top of each other. The microphotographs in (**C**) depict the binding of the transcription factor Sox2 to many nuclear territories, which is a measure of stemness. - Bars, 50 µm. Sox2, SRY (Sex Determining Region Y)-Box 2. (**D**) BrdU ELISA using 1 µM atRA, 1 µM CD3254 or increasing concentrations of the RAR pan agonist CD0270. The mean of eight independent values and the standard deviations are shown. For the sake of clarity, only statistical comparisons to the DMSO control and within the A/X group are shown (*, $p < 0.05$; **, $p < 0.001$). A/X, indicates co-application of 1 µM CD3254 and increasing concentrations of CD0270 (0.01, 0.1 and 1 µM).

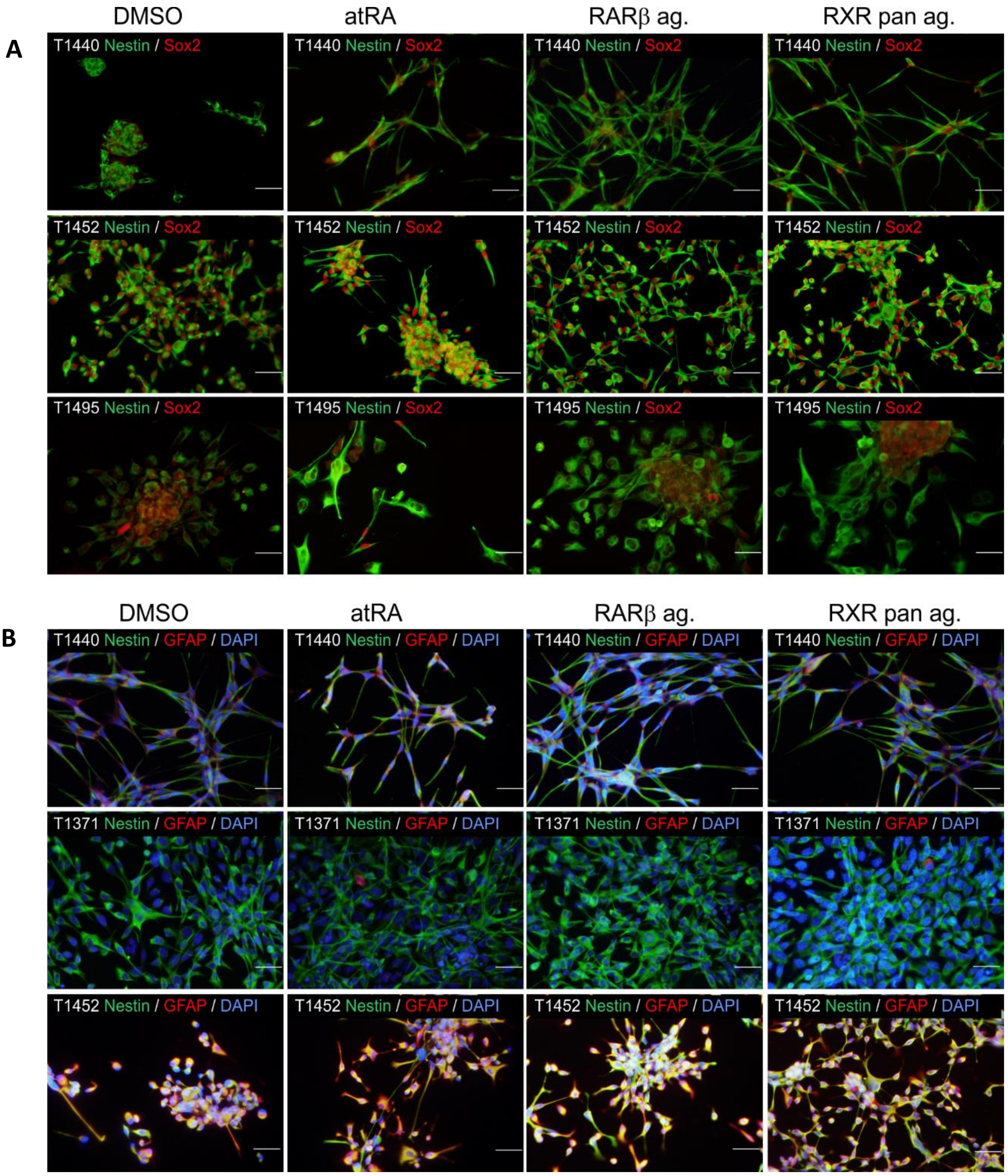

Figure S2. Effects of retinoids on the expression of neural proteins. (**A**) Immunocytochemistry analysis of SLGC lines on day d4 after treatment with ligands. Double stain with the antibody pairs mouse-anti-Nestin/goat-anti-mouse-DyLight© and rabbit-anti-Sox2/goat-anti-rabbit-Cy3. (**B**) Similar experiment as in (**A**) using the antibody pairs mouse-anti-Nestin/goat-anti-mouse-DyLight© and rabbit-anti-GFAP/goat-anti-rabbit-Cy3. DAPI nuclear counterstain was performed for all assays but omitted from the microphotographs in (**A**). Bars, 50 μm; Sox2, SRY (Sex Determining Region Y)-Box 2; GFAP, glial fibrillary acidic protein; DAPI, 4′,6-Diamidino-2-phenylindol.

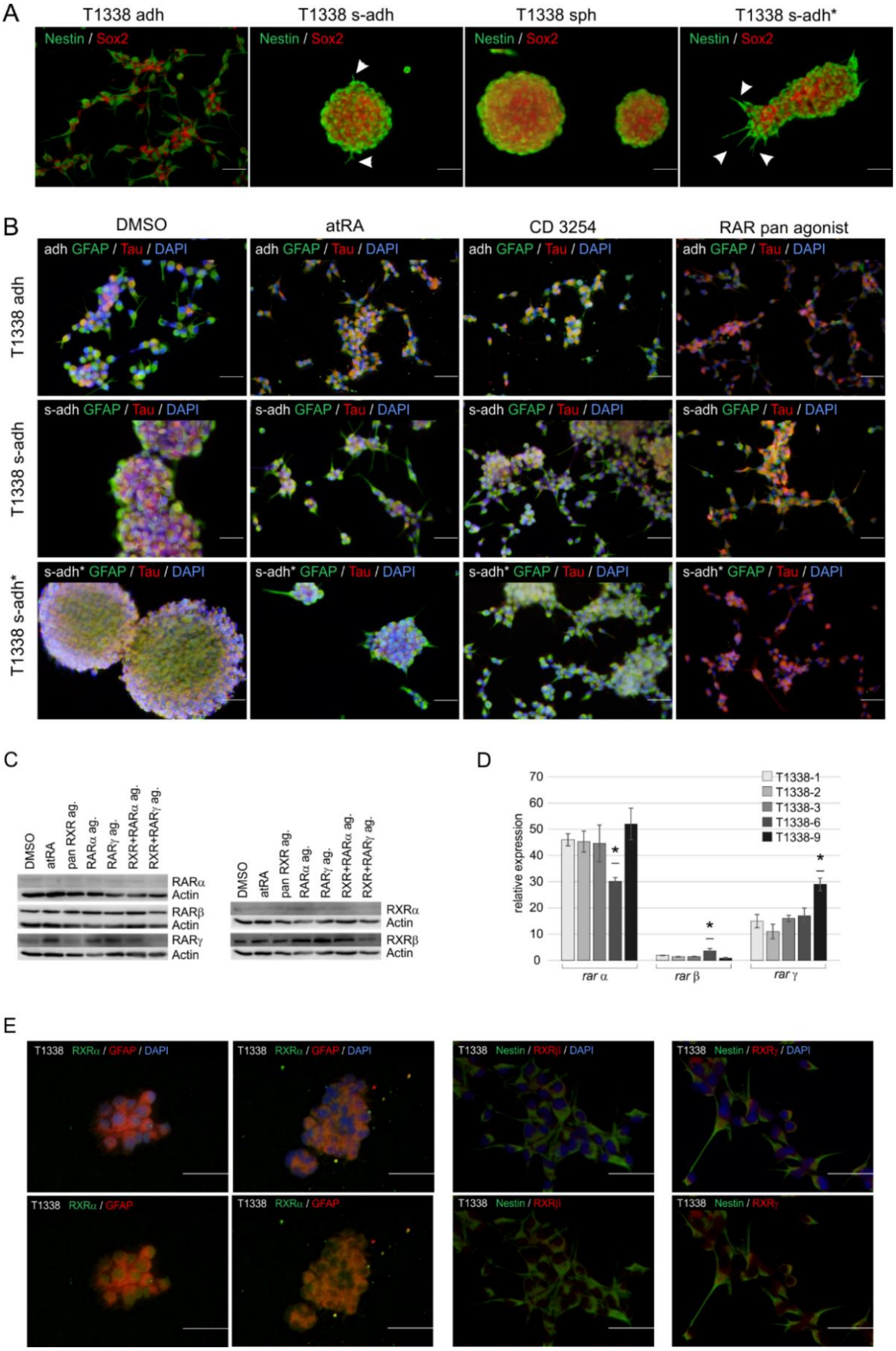

Figure S3. Characteristic of T1338 subclones. (**A**) Comparison of the stemness states of distinct T1338 subclones. The antibody combinations mouse-anti-CD133/goat-anti-mouse DyLight© and rabbit-anti-Sox2/goat-anti-rabbit Cy3 were used. Nuclear counterstain was performed with DAPI (4',6-Diamidino-2-phenylindol). Bars, 50 µm. Sox2, SRY (Sex Determining Region Y)-Box 2. The T1338 clones displayed primarily adherent (adh), semi-adherent (s-adh) or spherical (sph) growth; s-adh and s-adh* represent distinct T1338 subclones. Adherent aggregates are characterized by the formation of cellular appendages (white arrowheads), which contact the growth substrate and are missing in the floating aggregates. (**B**) Immunocytochemistry analysis of T1338 clones on day d4 after treatment with 1 µM of atRA, the pan-RXR agonist CD3254 or the RAR panagonist CD0270. Double stain with the antibody

pairs mouse-anti-GFAP/goat-anti-mouse-DyLight© and rabbit-anti-Tau/goat-anti-rabbit-Cy3 and DAPI nuclear counterstain. (**C**) Western blot analyses using the antibodies indicated. The loading control Actin is shown below the corresponding blots. Treatment was with 1 µM atRA (*all-trans* retinoic acid), CD3254 (RXR panagonist), CD1556 (RARα agonist), CD2664 (RARγ agonist) or a combination of the ligands. (**D**) qRT-PCR analysis indicating the expression of RARα, RARβ, and RARγ mRNAs in distinct T1338 clones and upregulation by atRA. *, indicates that mRNA expression is significantly different from that in other T1338 subclones. (**E**) Immunocytochemistry analysis using the antibody pairs indicated. DAPI nuclear counterstain was performed for all assays but omitted from some of the microphotographs depicting the staining for RXRα/GFAP. Bars, 50 µm. – Sox2, (Sex Determining Region Y)-Box 2; GFAP, glial fibrillary acidic protein; DAPI, 4',6-Diamidino-2-phenylindol.

```
A  RARA WT     TCGCCACCCCCTCTACCCCGCATCTACAAGCCTTGCTTTGTCTGTCAGGACAA
   cl D1α      TCGCCACCCCCTCTACC_CGGGACCTAA__________TTTGTCTGTCAGGACAA

   RARA WT     TCGCCACCCCCTCTACCCCGC - ATCTACAAGCCTTGCTTTGTCTGTCAGGACAA
   cl D11.1α   TCGCCACCCCCTCTACCCCGCAATCTACAAGCCTTGCTTTGTCTGTCAGGACAA
   cl B3α      TCGCCACCCCCTCTACCCCGCAATCTACAAGCCTTGCTTTGTCTGTCAGGACAA
   cl D4α      TCGCCACCCCCTCTACCCCGCAATCTACAAGCCTTGCTTTGTCTGTCAGGACAA
   cl B5α      TCGCCACCCCCTCTACCCCGCAATCTACAAGCCTTGCTTTGTCTGTCAGGACAA
   cl F3α      TCGCCACCCCCTCTACCCCGCAATCTACAAGCCTTGCTTTGTCTGTCAGGACAA
   cl G3α      TCGCCACCCCCTCTACCCCGCAATCTACAAGCCTTGCTTTGTCTGTCAGGACAA
   cl C2α      TCGCCACCCCCTCTACCCCGCAATCTACAAGCCTTGCTTTGTCTGTCAGGACAA
   cl D2α      TCGCCACCCCCTCTACCCCGCAATCTACAAGCCTTGCTTTGTCTGTCAGGACAA
   cl F6α      TCGCCACCCCCTCTACCCCGCAATCTACAAGCCTTGCTTTGTCTGTCAGGACAA
   cl G2α      TCGCCACCCCCTCTACCCCGCAATCTACAAGCCTTGCTTTGTCTGTCAGGACAA
   cl G4α      TCGCCACCCCCTCTACCCCGCAATCTACAAGCCTTGCTTTGTCTGTCAGGACAA
   cl E2α      TCGCCACCCCCTCTACCCCGCTATCTACAAGCCTTGCTTTGTCTGTCAGGACAA

B  RARB WT     AAGCCCCCCATCTCCACTTCC -T - - CCCCCTCGAGTGTACAAACCCTGCTTCGTCTGCCAGG
   cl C2β      AAGCCCCCCATCTCCACTTCCCTGCTTCGTCTGCCAGGACAAACCCTGCTTCGTCTGCCAGG
   cl C2β      AAGCCCCCCATCTCCACTTCC_T__CCCCCTCGA__ GTACAAACCCTGCTTCGTCTGCCAGG
   cl C2β      AAGCCCCCCATCTCCACTTCC_T________________________ CCCTGCTTCGTCTGCCAGG
   cl C2β      AAGCCCCCCA_____________________________________AACCCTGCTTCGTCTGCCAGG

   RARB WT     AAGCCCCCCATCTCCACTTCCTCCCCCTCGAGTGTACAAACCCTGCTTCGTCTGCCAGG
   cl D3/3β    AAGCCCCCCATCTCCACTTCCTCCCCCTCG_GTGTACAAACCCTGCTTCGTCTGCCAGG
   cl D5/3β    AAGCCCCCCATCTCCACTTCCTCCCC______________________ CTTCGTCTGCCAGG

   RARB WT     CAATTGAAACACAGAGCACCAGCTCTGAGGAACTCGTCCCAAGCCCCCCATCTCCACTT
   cl D8β      CAATTGAAACACAGA ____________________________________________
   cl D8β      CAATTGAAACACAGAGCACCAGCTCTGAGGAACTCGTCCCAAGCCCCCCATCTCCACTT

   RARB WT     CCTCCCCCTCGAGTGTACAAACCCTGCTTCGTCTGCCAGGACAAATCATCAGGGTACCA
   cl D8β      ___________________________________________________________
   cl D8β      CCTCCCCCTCGAGTGTACAAACCCTGCTTCGTCTGCCAGGACAAATCATCAGGGTACCA

   RARB WT     CTTAGGGGTCAGCGCCTGTGAGGGATGTAAG
   cl D8β      ____ GGGGTCAGCGCCTGTGAGGGATGTAAG
   cl D8β      CTTAGGGGTCAGCGCCTGTGAGGGATGTAAG

C  RARG WT     TCGCCCCCTCCGCCTCCTCGGGTCTACAAGCCATGCTTCGTGTGCAATGA
   cl D1γ      TCGCCCCCTCCGCCTCCTC___GTCTACAAGCCATGCTTCGTGTGCAATGA
   cl D1γ      TCGCCCCCTCCG______________________________ CAATGA
   cl D1γ      TCGCCCCCTCCGCCTCCTCGG__________CCATGCTTCGTGTGCAATGA
   cl F2γ      TCGCCCCCTCCGCCTC_______________ CCATGCTTCGTGTGCAATGA

   RARG WT     TCGCCCCCTCCGCCTCCTC - - GGGTCTACAAGCCATGCTTCGTGTGCAATGA
   cl F11γ     TCGCCCCCTCCGCCTCTTCGGGGGTTTACAAGCCATGCTTCGTGTGCAATGA

   RARG WT     TCGCCCCCTCCGCCTCCTC - GGGTCTACAAGCCATGCTTCGTGTGCAATGA
   cl D1γ      TCGCCCCCTCCGCCTCCTCGGGGTCTACAAGCCATGCTTCGTGTGCAATGA
   cl F2γ      TCGCCCCCTCCGCCTCCTCGGGGTCTACAAGCCATGCTTCGTGTGCAATGA
   cl F11γ     TCGCCCCCTCCGCCTCCTCGGGGTCTACAAGCCATGCTTCGTGTGCAATGA
```

Figure S4. Mutations introduced in the RARA, RARB or RARG genes by CRISPR/Cas9-mediated editing. Alignment of the nucleotide sequences. The PAM (protospacer adjacent motif) is indicated in blue; the single guide (sg) RNA binding region is underlined. Deleted nucleotides are represented by lines; newly inserted nucleotides are shown in red. In the cases in which distinct edited sequences were identified in the same cell population, the various sequences are shown. Wildtype sequences with gaps are shown above edited sequences with insertions. Please note that the selected PAM sequence reads NGG and is located on the antisense strand in the cases of the RARA, RARB or RARG genes; for sake of clarity, the coding strands are depicted in the panels. The symbol behind the clone number indicates the targeted RAR isotype gene.

double editing approach RARA plus RARG

RARG WT	TCGCCCCCTCCGCCT**CCT**CGGGTCTACAAGCCATGCTTCGTGTGCAATGA
cl B4 a **g***	TCGCCCCCTCCGCCT**CCT**CGG-nnn
cl C2 **a*g***	TCGCCCCCTCCGCCT**CCT**CGG-nnn
cl D1 **a*g***	TCGCCCCCTCCGCCT**CCT**CGG-nnn
cl D2 **a*g***	TCGCCCCCTCCGCCT**CCT**CG-nnn
cl F1 a **g***	TCGCCCCCTCCGCCT**CCT**CGGGT-nnn
cl F6 **a*g***	TCGCCCCCTCCGCCT**CCT**CGGG-nnn
cl G2 **a***g	TCGCCCCCTCCGCCT**CCT**CGGGTCTACAAGCCATGCTTCGTGTGCAATGA
cl G4 **a*g***	TCGCCCCCTCCGCCT**CCT**CGG-nnn

double editing approach RARB plus RARG

RARG WT	TCGCCCCCTCCGCCT**CCT**CGGGTCTACAAGCCATGCTTCGTGTGCAATGA
cl B3 **b*g***	TCGCCCCCTCCGCCT**CCT**CGG-nnn
cl B9 **b*g***	TCGCCCCCTCCGCCT**CCT**CGGG-nnn
cl C5 b **g***	TCGCCCCCTCCGCCT**CCT**CGG-nnn
cl E8 b **g***	TCGCCCCCTCCGCCT**CCT**C-nnn
cl F8 b **g***	TCGCCCCCTCCGCCT**CCT**C-nnn
cl G9 b **g***	TCGCCCCCTCCGCCT**CCT**CGG-nnn

double editing approach RARA plus RARB

RARB WT	AAGCCCCCCATCTCCACTT**CCT**CCCCCTCGAGTGTACAAACCCTGCTTCGTCTGCCAGG
cl B3 **a***b	AAGCCCCCCATCTCCACTT**CCT**CCCCCTCG-nnn
cl D4 **a***b	AAGCCCCCCATCTCCACTT**CCT**CCCCCTCG-nnn
cl B5 **a***b	AAGCCCCCCATCTCCACTT**CCT**CCCCCTCGAG-nnn
cl E2 **a***b	AAGCCCCCCATCTCCACTT**CCT**CCCCCTCGAGTGTACAAACCCTGCTTCGTCTGCCAGG
cl F3 **a***b	AAGCCCCCCATCTCCACTT**CCT**CCCCCTC-nnn
cl G3 **a***b	AAGCCCCCCATCTCCACTT**CCT**CCCCCTCG-nnn
cl H3 a b	AAGCCCCCCATCTCCACTT**CCT**CCCCCTCGAGTG-nnn
cl H5 a b	AAGCCCCCCATCTCCACTT**CCT**CCCCCTCGA-nnn

Figure S5. Editing of two distinct RAR genes is possible in T1338 cells. Alignment of RARB or RARG nucleotide sequences for editing approaches "a g" (RARA plus RARG), "b g" (RARB plus RARG) and "a b" (RARA plus RARB). Red letters (a, b, or g) indicate the presence of mutations, black letters (a, b, or g) indicate the wildtype status. When sequencing of the PCR products revealed multiple edits, this is indicated by *. Only a subgroup of clones was analyzed by TA cloning followed by Sanger sequencing. In all other cases, the edited sequence was not deduced in detail (-nnn), except for the cases in which the mutations were homozygous. Homozygous mutations were observed for >98 % of the RARA genes and resulted in the insertion of an additional A nucleotide in >90% of the clones (see details in Figure S4). Therefore, the alignments of the RARA sequences are not depicted in the panels depicting the results of the double editing approaches. Please note that the selected PAM sequences read NGG and are located on the antisense strand in the cases of the RARA, RARB or RARG genes; for sake of clarity, the coding strands are depicted in the panels.

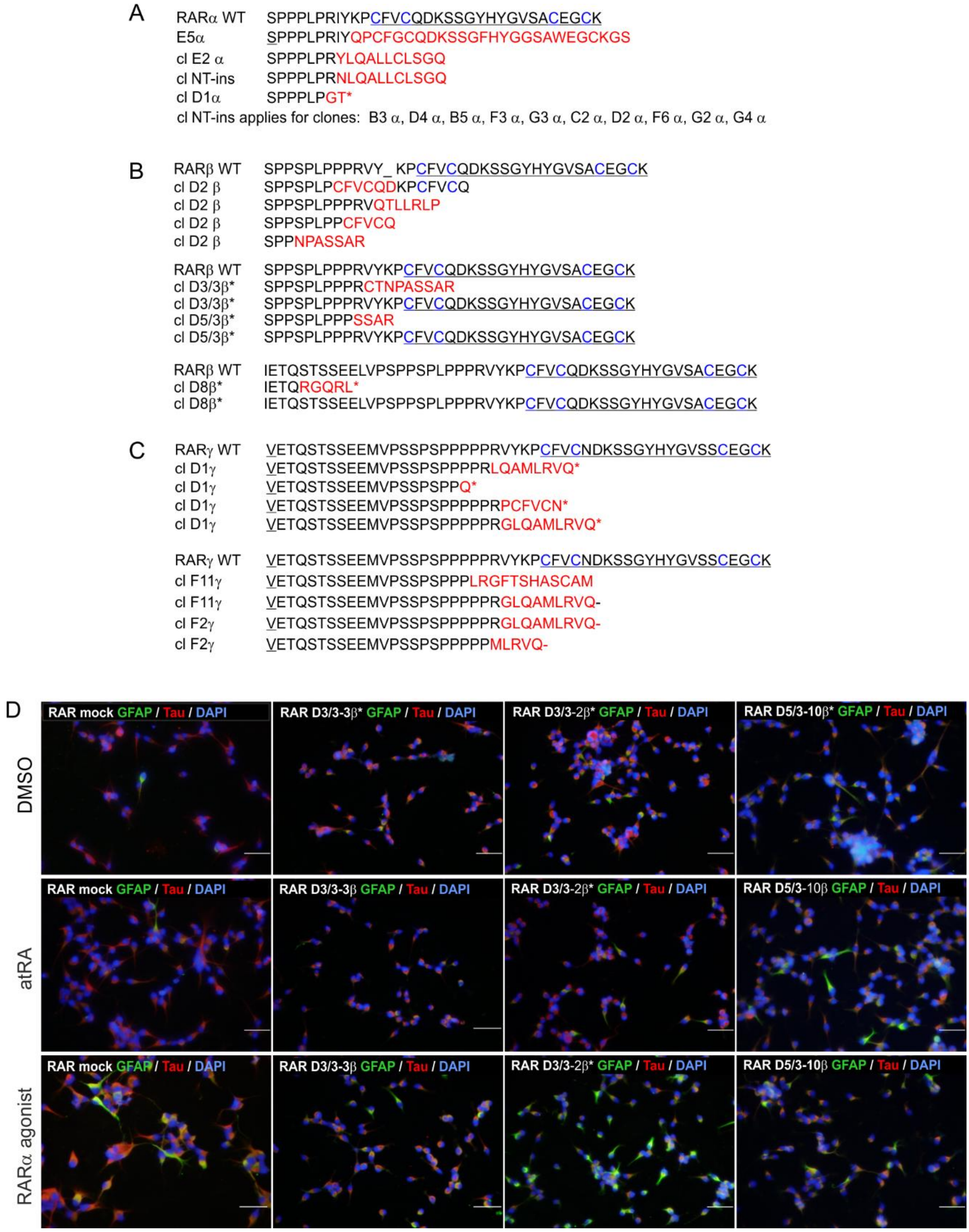

Figure S6. CRISPR/Cas9-mediated editing of the RAR genes. (**A,B,C)** Alignment of the deduced amino acid sequences (one-letter code) of the clones (cl) indicated. The two pairs of cysteine residues (C-xx-C and C-EG-C) that coordinate zinc binding to the first zinc finger are indicated in blue; the amino acid sequence corresponding to the first zinc finger is underlined; mutations are indicated in red. Interruptions of the wildtype (WT) sequences are shown in the cases in which insertions occurred in the edited sequences. In cases of clones in which more than one mutation occurred (or residual wildtype sequences were present), the various amino acid sequences are shown**.** NT-ins, insertion of the same single nucleotide; β*, indicates monoallelic editing of the RARB gene; *, a premature stop codon was present. (**D**) Differentiation after treatment with 1 μM atRA or 1 μM CD1556 (RARα agonist). Immunocytochemistry analyses using the antibody pair mouse-anti-GFAP/rabbit-anti-Tau and DAPI nuclear counterstain. Bars, 50 μm. – GFAP, glial fibrillary acidic protein; Tau, microtubule-associated neuronal protein.

```
A   RXRA WT     GCAAGCACTATGGAGTGTACAGCTGCGAGGGGTGCAAGGGCTTCTT
    cl F2α      GCAAGCACTATGGAGTGTAC_CCT_ CGAGGGGTGCAAGGGCTTCTT

    RXRA WT     GCAAGCACTATGGAGTGTACAGCT- GCGAGGGGTGCAAGGGCTTCTT
    cl E8α      GCAAGCACTATGGAGTGTACAGCTTGCGAGGGGTGCAAGGGCTTCTT
    cl C10α     GCAAGCACTATGGAGTGTACAGCTTGCGAGGGGTGCAAGGGCTTCTT

    RXRA WT     GCAAGCACTATGGAGTGTACAGCT- GCGAGGGGTGCAAGGGCTTCTT
    cl D5α      GCAAGCACTATGGAGTGTACAGCTTGCGAGGGGTGCAAGGGCTTCTT
    cl D5α      GCAAGCACTATGGAGTGTACA____TGCGAGGGGTGCAAGGGCTTCTT

    RXRA WT     GCAAGCACTATGGAGTGTACAGCT- GCGAGGGGTGCAAGGGCTTCTT
    cl E10α     GCAAGCACTATGGAGTGTACAGCTTGCGAGGGGTGCAAGGGCTTCTT
    cl E10α     GCAAGCACTATGGAGTGTACA__ T_GCGAGGGGTGCAAGGGCTTCTT

    RXRA WT     GCAAGCACTATGGAGTGTACAGCT- GCGAGGGGTGCAAGGGCTTCTT
    cl F4α      GCAAGCACTATGGAGTGTACAGCTTGCGAGGGGTGCAAGGGCTTCTT
    cl F4α      GCAAGCACTATGGAGTGTACAGC_____GAGGGGTGCAAGGGCTTCTT
    cl F4α      GCAAGCACTATGGAGTGTACAGCT__ CGAGGGGTGCAAGGGCTTCTT

B   RXRB WT     GCAAACACTACGGGGTTTACAGCTGTGAGGGTTGCAAGGGCTTCTTCAAACGCAC
    cl E5β      GCAAACACTACGGGGTTTACAGCTGTGAGGGTT ______________________CAC
    cl E11β     GCAAACACTACGGGGTTTACAGCTGTGAGGGTGGCAAGGGCTTCTTCAAACGCAC
    cl E9β      GCAAACACTACGGGGTTTACAGCTGTGAGGGTTGCAGGGGCTTCTTCAAACGCAC
    cl E9β      GCAAACACTACGGGGTTTACAGCTGTGAGGGTTGTAAGGGCTTCTTCAAACGCAC

    RXRB WT     GCAAACACTACGGGGTTTACAGCTGTGAGGG- TTGCAAGGGCTTCTTCAAACGCAC
    cl C11β     GCAAACACTACGGGGTTTACAGCTGTGAGGGTTTGCAAGGGCTTCTTCAAACGCAC
    cl G3β      GCAAACACTACGGGGTTTACAGCTGTGAGGGTTTGCAAGGGCTTCTTCAAACGCAC

C   RXRG WT     GAAAGCACTACGGGGTATACAGTTGTGAAGGCTGCAAAGGGTT
    cl C8γ      GAAAGCACTACGGGGTATACAGTTG__ AAGGCTGCAAAGGGTT
    cl D7γ      GAAAGCACTACGGGGTA_________ TGAAAGGCTGCAAAGGGTT
    cl D7γ      GAAAGCACTACGGGGTATAGGTATACAGTTGAAGGCTGCAAAGGGTT

D   RXRA WT     GCAAGCACTATGGAGTGTACAGCT- GCGAGGGGTGCAAGGGCTTCTT
    cl H6αγ     GCAAGCACTATGGAGTGTACAGCTTGCGAGGGGTGCAAGGGCTTCTT
    cl C5αγ     GCAAGCACTATGGAGTGTACAGCTTGCGAGGGGTGCAAGGGCTTCTT

    RXRG WT     GAAAGCACTACGGGGTATACAGTTGTGAAGGCTGCAAAGGGTTCTTCA
    cl C5αγ     GAAAGCACTACGGGGTATACAGTTGTGAAGGCTGCAAAGGGTTCTTCA
    cl C5αγ     GAAAGCACTACGGGGTATACA___ GTGAAAGGCTGCAAAGGGTTCTTC

    RXRG WT     GAAAGCACTACGGGGTATACAGTTGTGAAGGCTGCAAAGGGTTCTTCA
    cl H6αγ     GAAAGCACTACGGGGTA______________________________
    cl H6αγ     GAAAGCACTACGGGGTATACAGTT__________ GGCAAAGGGTTCTTCA
    cl H6 C9αγ  GAAAGCACTACGGGGTATACGTT ____________ GCAAAGGGTTCTTCA
    cl H6 C9αγ  GAAAGCACTACGGGGTA_______________________________
    cl H6 F7αγ  GAAAGCA______________ AAGGCTGCAAAGGGTTCTTCA
    cl H6 C4αγ  GAAAGCACTACGGGGGATACGGGTGCTA___________ AGGTTCTTCA
    cl H6 C4αγ  GAAAGCACTACGGGGTATACGTT _____________GCAAAGGGTTCTTCA
    cl H6 C4αγ  GAAA ______________________ AAGGCTGCAAAGGGTTCTTCA

    RXRG WT     GAAAGCACTACGGGGTATACAGTTG - - - TGAAGGCTGCAAAGGGTTCTTCAAGA
    cl H6αγ     GAAAGCACTACGGGGTATACAAACCCTTTGAAGGCTGCAAAGGGTTCTTCAAGA
```

Figure S7. Mutations introduced in the RXRA, RXRB or RXRG genes by CRISPR/Cas9-mediated editing. (**A,B,C**) Clones with *knockouts* in either the RXRA, RXRB, or RXRG genes. (**D**) Clones with *knockouts* in RARA plus RXRG. Alignment of the nucleotide sequences. The PAM (protospacer adjacent motif) is indicated in blue; the single guide (sg) RNA binding region is underlined. Deleted nucleotides are represented by lines; newly inserted nucleotides are shown in red. In the cases in which distinct edited sequences were identified in the same cell population, the various sequences are shown. Wildtype sequences with gaps are shown above edited sequences with insertions. The symbol behind the clone number indicated the targeted RXR gene.

A

RXRα WT CAICGDRSSGKHYGVYSCEGCKGFFKRTVRKDLTYTCRDNKDCLIDKRQRNRCQYCRY
cl F2 α CAICGDRSSGKHYGVYPRGVQLLQADGAQGPDLHLPRQQGLPD*
cl D5 α CAICGDRSSGKHYGVYMRGVQGLLQADGAQGPDLHLPRQQGLPD*
cl D5 α CAICGDRSSGKHYGVYSLRGVQGLLQADGAQGPDLHLPRQQGLPD*

RXRα WT CAICGDRSSGKHYGVYSCEGCKGFFKRTVRKDLTYTCRDNKDCLIDKRQRNRCQYCRY
cl NT-ins CAICGDRSSGKHYGVYSLRGVQGLLQADGAQGPDLHLPRQQGLPD*
cl C5 αγ CAICGDRSSGKHYGVYSLRGVQGLLQADGAQGPDLHLPRQQGLPD*
cl H6 αγ CAICGDRSSGKHYGVYSLRGVQGLLQADGAQGPDLHLPRQQGLPD*
cl NT-ins: applies for clones E6 α, E8 α, C10 α, E10 α

RXRα WT CAICGDRSSGKHYGVYSCEGCKGFFKRTVRKDLTYTCRDNKDCLIDKRQRNRCQYCRY
cl F4 α CAICGDRSSGKHYGVYSSRGARASSSGRCART*
cl F4 α CAICGDRSSGKHYGVYS_EGCKGFFKRTVRKDLTYTCRDNKDCLIDKRQRNRCQYCRY
cl F4 α CAICGDRSSGKHYGVYSLRGVQGLLQADGAQGPDLHLPRQQGLPD*
cl F4 α CAICGDRSSGKHYGVYSSRGARASSSGRCART*

B

RXRβ WT CAICGDRSSGKHYGVYSCEGCKGFFKRTIRKDLTYSCRDNKDCTVDKRQRNRCQYCRY
cl E 5β CAICGDRSSGKHYGVYSCEGSPSAKTLHTLAGTTKTAQWTSASGTAVSTAAIRSAWPLA*
cl G 3β CAICGDRSSGKHYGVYSCEGLQGLLQTGGPQRPYILLPGQQRLHSGQAPAEPLSVL-----

cl E9 β CAICGDRSSGKHYGVYSCEGCRGFFKRTIRKDLTYSCRDNKDCTVDKRQRNRCQYCRY
cl E9 β CAICGDRSSGKHYGVYSCEGCKGFFKRTIRKDLTYSCRDNKDCTVDKRQRNRCQYCRY

C

RXRg WT CAICGDRSSGKHYGVYSCEGCKGFFKRTIRKDLIYTCRDNKDCLIDKRQRNRCQYCRYQKC
cl C8 γ CAICGDRSSGKHYGVYS*
cl D7 γ CAICGDRSSGKHYGV*
cl C5 αγ CAICGDRSSGKHYGVYSERLQRVL*
cl C5 αγ CAICGDRSSGKHYGVYSCEGCKGFFKRTIRKDLIYTCRDNKDCLIDKRQRNRCQYCRYQKC

RXRg WT CAICGDRSSGKHYGVYS_CEGCKGFFKRTIRKDLIYTCRDNKDCLIDKRQRNRCQYCRYQKC
cl H6 αγ CAICGDRSSGKHYGV_________VVLLQ_______ YTCRDNKDCLIDKRQRNRCQYCRYQKC
cl H6 αγ CAICGDRSSGKHYGVYKPFEGCKGFFKRTIRKDLIYTCRDNKDCLIDKRQRNRCQYCRYQKC
cl H6 αγ CAICGDRSSGKHYGVYS_C____KGFFKRTIRKDLIYTCRDNKDCLIDKRQRNRCQYCRYQKC

D

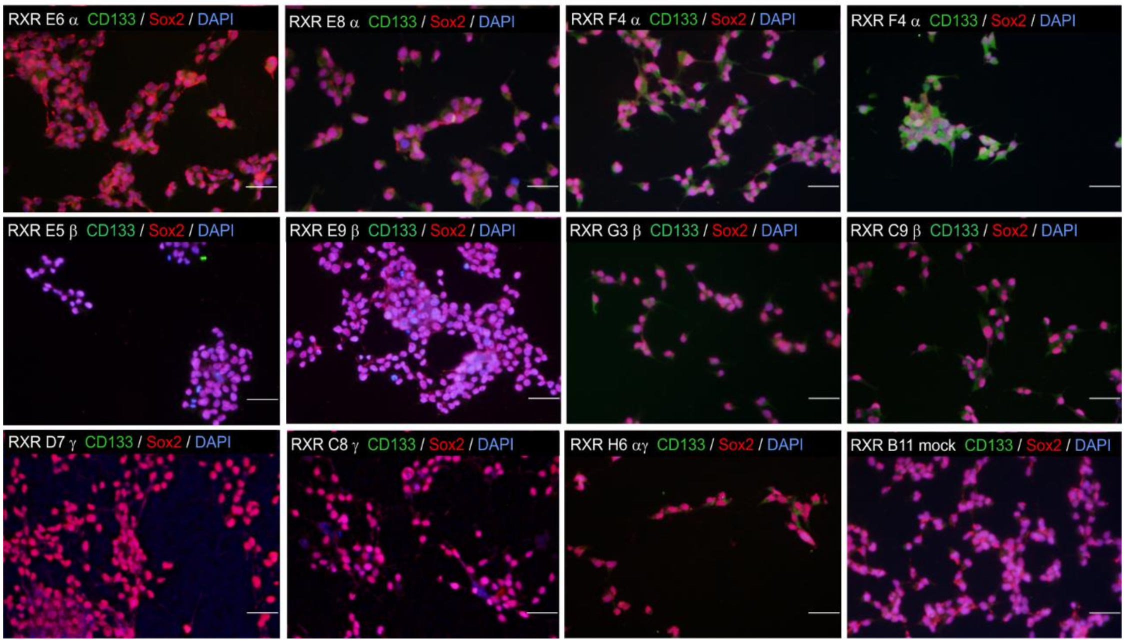

Figure S8*. CRISPR/Cas9-mediated editing of the RXR genes.* (**A**,B,**C**) Alignment of the deduced amino acid sequences (one-letter code) of the clones (cl) indicated. The two pairs of cysteine residues (C-xx-C and C-EG-C) that coordinate zinc binding to the first zinc finger are indicated in blue; the amino acid sequence corresponding to the first zinc finger is underlined; mutations are indicated in red. Interruptions of the wildtype (WT) sequences are shown in the cases in which insertions occurred in the edited sequences. In the cases of clones in which more than one mutation occurred (or residual wildtype sequences were present), the various amino acid sequences are shown**.** NT-ins, clones with an insertion of the same single nucleotide; *, premature stop codon. (**D**) Immunocytochemistry analyses of the stemness state of the edited clones using antibodies against Sox2 and CD133 and DAPI nuclear counterstain. Bars, 50 μm.

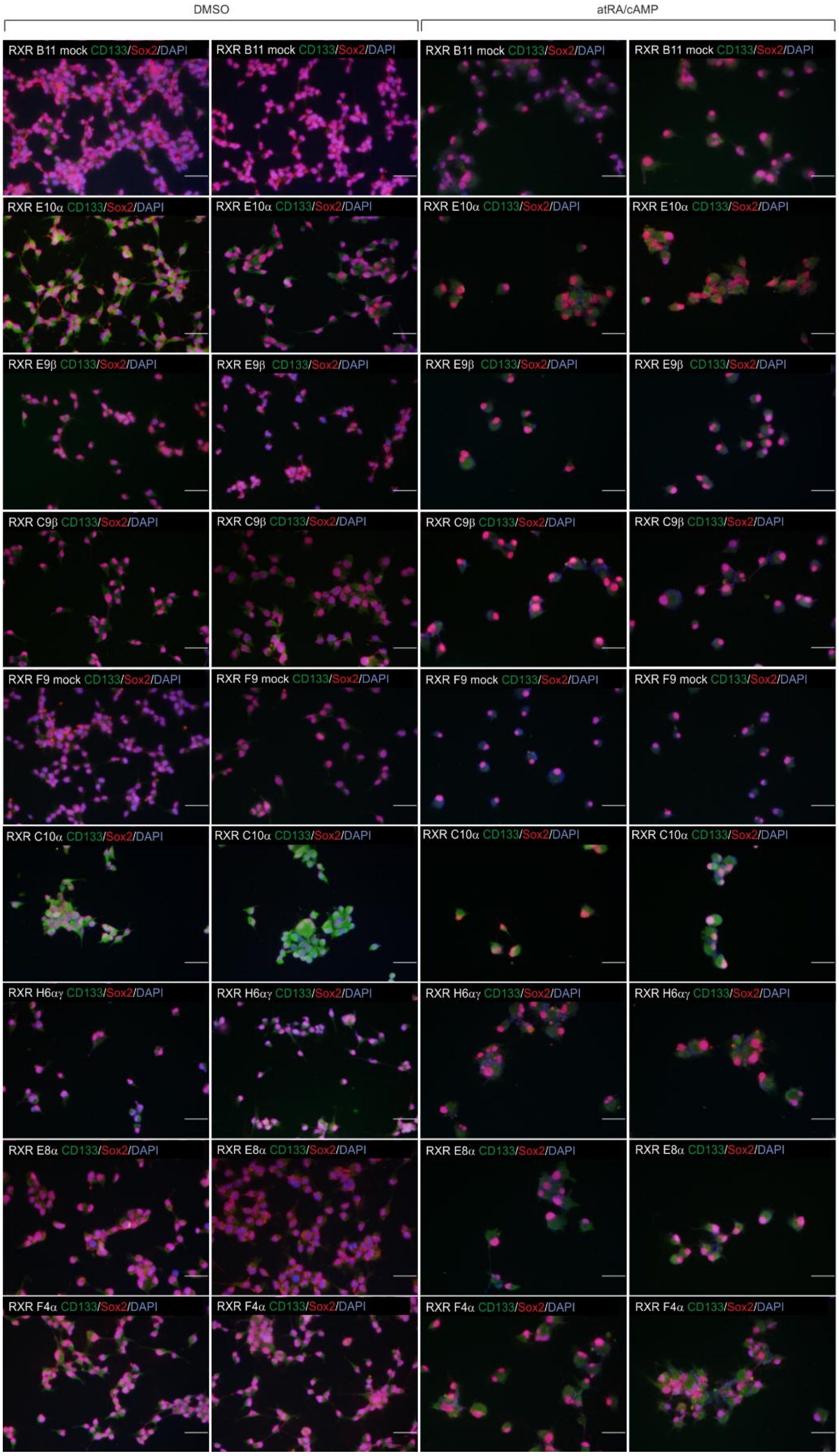

Figure S9. Degree of stemness after treatment with atRA and cAMP. Immunocytochemistry analysis of T1338 clones harboring edited RXRA, RXRB, and/or RXRG genes. Double stains with the antibody pairs mouse-anti-CD133/goat-anti-mouse-DyLight© and rabbit-anti-Sox2/goat-anti-rabbit-Cy3 and DAPI nuclear counterstain. Bars, 50 µm. Data from two biological replicates are shown. – Sox2, SRY (Sex Determining Region Y)-Box 2; cAMP, cyclic adenosine monophosphate; DAPI, 4′,6-Diamidino-2-phenylindol. The symbol behind the clone number indicated the targeted RXR gene.

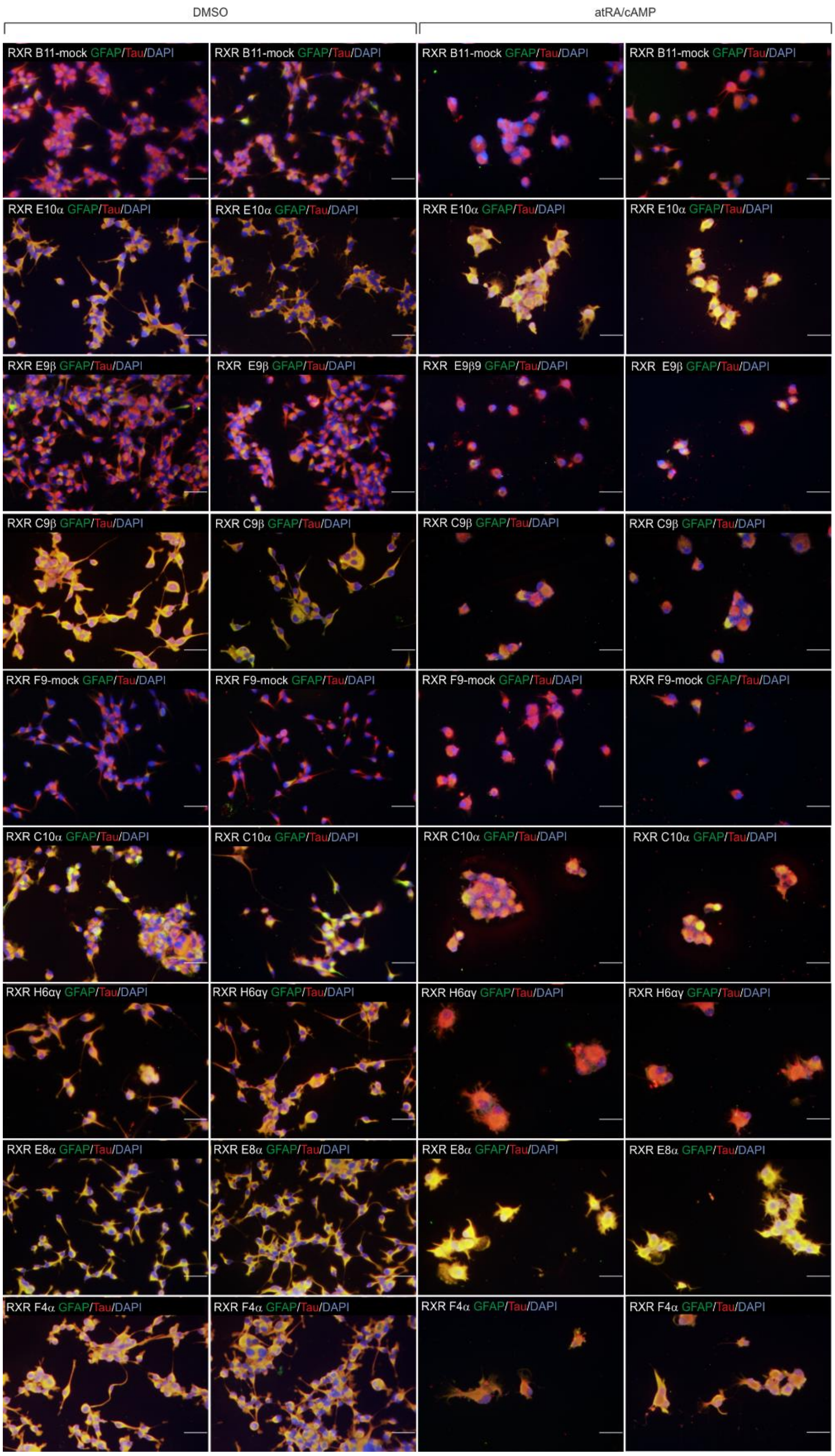

Figure S10. Capacity of atRA and cAMP to induce differentiation. Immunocytochemistry analysis of T1338 clones harboring edited RXRA, RXRB or RXRG genes. Double stains with the antibody pairs mouse anti-GFAP/goat-anti-mouse-DyLight© and rabbit-anti-Tau/goat-anti-rabbit-Cy3 and DAPI nuclear counterstain. Bars, 50 µm. Data from two biological replicates are shown – GFAP, glial fibrillary acidic protein; cAMP, cyclic adenosine monophosphate; DAPI, 4′,6-Diamidino-2-phenylindol. The symbol behind the clone number indicated the targeted RXR gene.

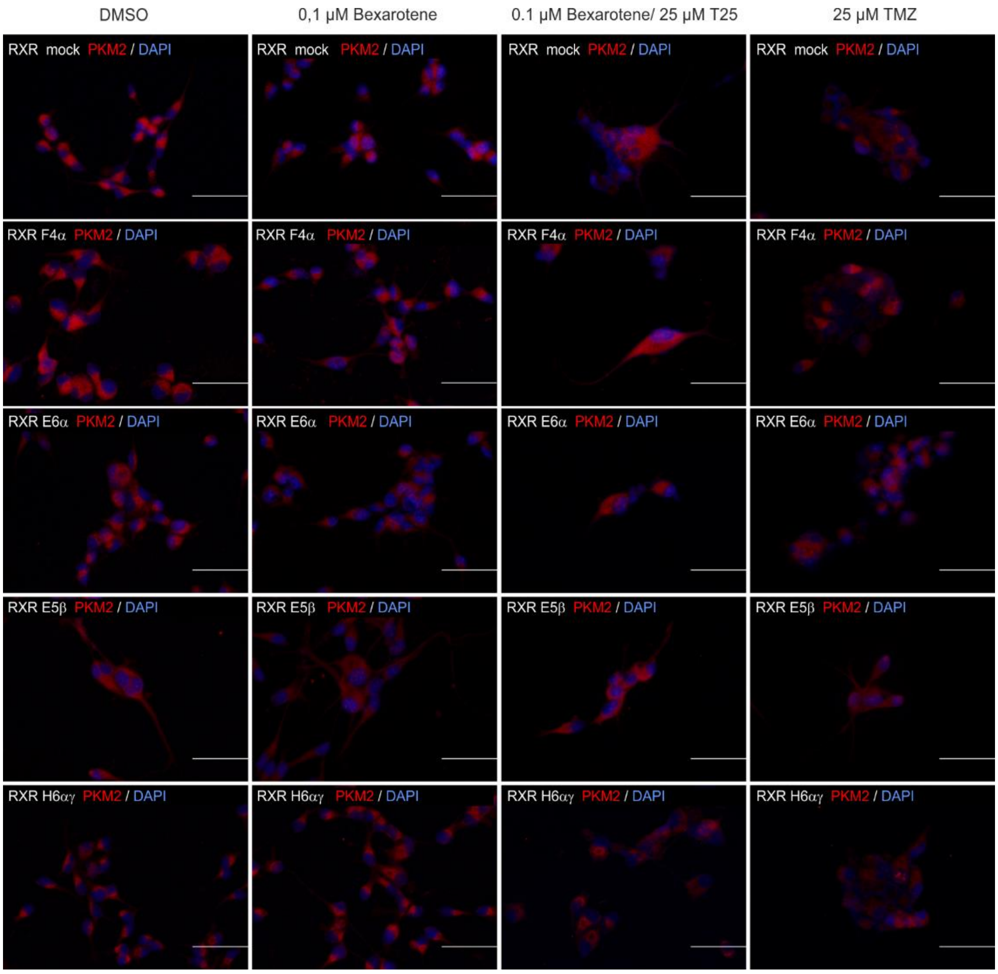

Figure S11. Expression of PKM2. Immunoccytochemistry analysis investigating the expression of the PKM2 on day d5 after treatment with Bexarotene and/or 25 µM Temozolomide (T25) and DAPI nuclear counterstain. The mock edited clone (RXR B11) and four clones harboring edited RXRA, RXRB and/or RXRG genes were analyzed. Bars, 50 µm. – DAPI, 4′,6-Diamidino-2-phenylindol.

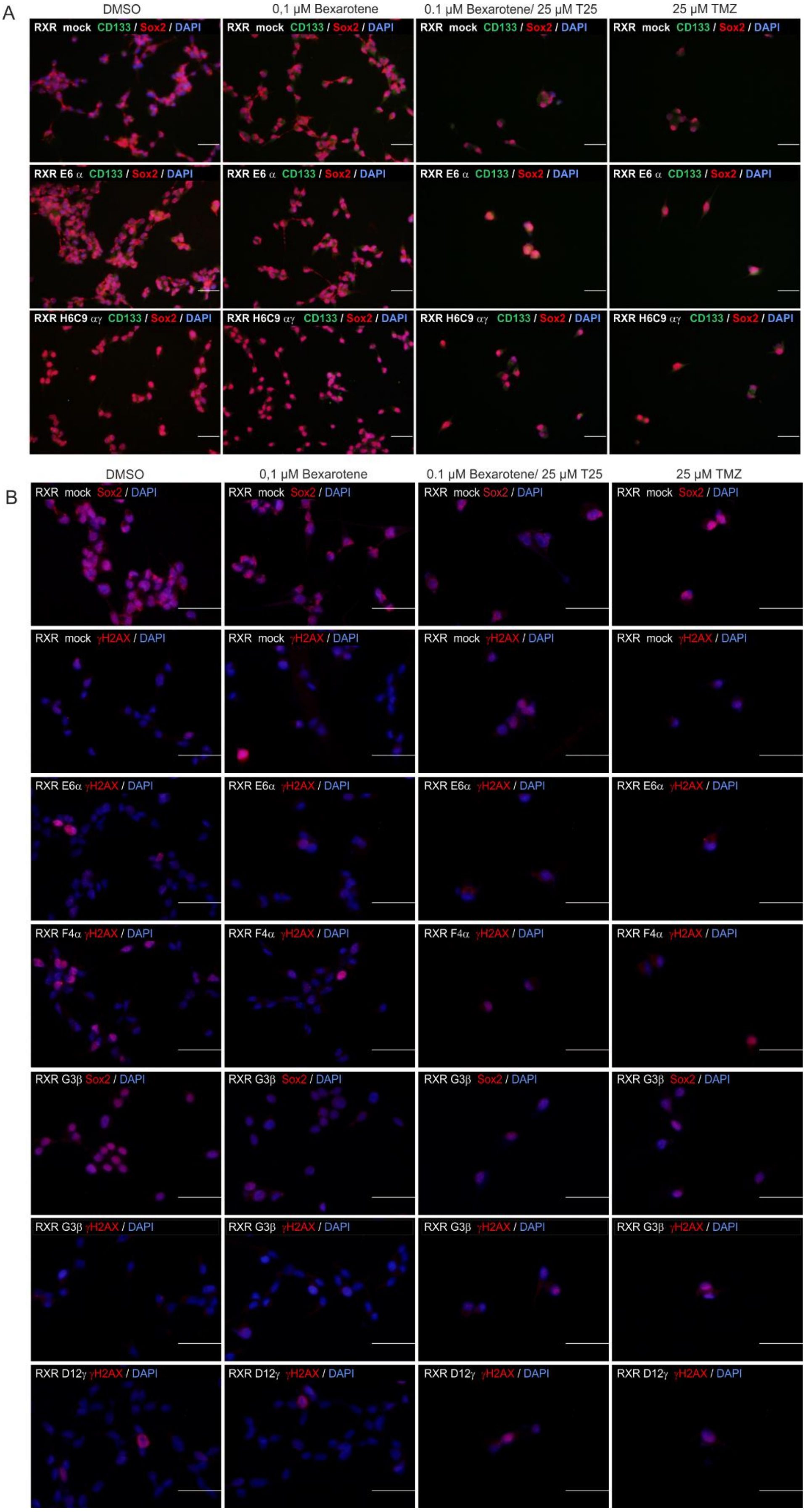

Figure S12. Degree of stemness and induction of the phosphorylation of the histone variant H2AX (γH2AX) after treatment with TMZ and/or bexarotene. Immunocytochemistry analyses were performed on day d5 after treatment.

(**A**) The degree of stemness was analyzed using the antibody combination mouse-anti-CD133/rabbit-anti-Sox2, followed by DAPI nuclearcounter stain. Bars, 50 μm. (**B**) The phosphorylation of the histone variant H2AX was analyzed using the antibody combination mouse-anti-Nestin/rabbit-anti-γH2AX, followed by nuclear counterstain. The γH2AX signal and the DAPI nuclear counterstain are shown in the microphotographs; the Nestin signal was omitted for the sake of clarity. Importantly, the analysis in (B) was performed in parallel to an experiment investigating the degree of stemness, two examples of which (RXR B11 mock; RXR G3β) are shown in (B). Bars, 50 μm.
